# Complete mitochondrial genome assembly and comparison of *Camellia sinensis* var. *Assamica* cv. *Duntsa*

**DOI:** 10.3389/fpls.2023.1117002

**Published:** 2023-01-19

**Authors:** Jin Li, Han Tang, Hua Luo, Jun Tang, Ni Zhong, Lizheng Xiao

**Affiliations:** ^1^ Key Laboratory of Tea Science of Ministry of Education, National Research Center of Engineering and Technology for Utilization of Botanical Functional Ingredients, Co-Innovation Center of Education Ministry, Changsha, China; ^2^ Institute of Tea Research, Shaoyang Academy of Agricultural Sciences, Shaoyang, China; ^3^ Institute of Tea Research, Hunan Academy of Agricultural Sciences, Changsha, China

**Keywords:** *Camellia sinensis* var. *Assamica* cv. *Duntsa*, horizontal transfer, repeat sequence, genome size variation, mitochondrial genome, evolution, phylogenetic

## Abstract

*Camellia sinensis* var. *Assamica* cv. *Duntsa* (*C.duntsa*), a valuable *Theaceae* from Hunan Province, has been looked at as a precious tea resource by local farmers because of its economic and ecological value. Genomics study on *C.duntsa* is essential for the domestication and enhancement of tea tree varieties. In the present study, we used a hybrid approach based on Illumina and PacBio data to sequence and assemble the mitochondrial genome of *C.duntsa*. The mitochondrial genome of *C.duntsa* was estimated to be 1,081,996 base pairs (bp) and eighty-one genes consisting of one pseudogene, three ribosomal RNA (rRNA) genes, thirty transfer RNA (tRNA) genes, and forty-seven protein-coding genes (PCGs). Tetramer repetitions made up 43.90% of simple sequence repeats (SSRs). The codon usage bias of the *Theaceae* mitochondrial gene *atp9* was altered by mutation, but the codon usage of other genes was shaped by natural selection. Besides, there are eighteen gene-containing homologous regions between the chloroplast and mitochondrial genomes of *C. duntsa*.Some genomes including *atp8*, *cox1*, *cox3*, *nad7*, *nad9*, *rpl16*, *rpl2*, *rps19*, *rps4*, and *sdh4* are absent in the mitochondrial genome of several *Theaceae* plant. However, *C. duntsa* maintains these genes integrity and functionality. Another gene, *rps16*, is either lacking from the mitochondrial genome of *C. duntsa* or is present as a pseudogene. *C. duntsa* and *C. sinensis* (OM809792) are very similar, as shown by a collinear match across four species of *Theaceae*; the most conservative genes are *nad5*, *atp9*, *cox2*, *rps3*, *trnA-TGC*, *trnI-GAT*, *rrn18*, *trnV-GAC*, and *ccmFN*. Similarly, the genome’s phylogenetic trees revealed that *C. duntsa* was the sister species to *C. sinensis*. The results confirmed that the *C. duntsa* and *C. sinensis* (OM809792) mitochondrial genome underwent gene rearrangement.In general, our results shows that genomic information from organelles can help us understand plant phylogeny and can also be used to make molecular markers and study how genetic traits change over time. Our research will contribute to the population genetics and evolution of tea plant.

## 1 Introduction

The basic function of mitochondria in living cells is to transform biomass energy into chemical energy (Birky, 1995).Most seed plants inherit mitochondrial genetic information from the maternal line (Cusimano and Wicke, 2016).The size, sequence alignment, number of repeats, and structure of plant mitochondrial genomes are all very complex and different (Mower et al., 2012). Higher plants have mitochondrial genomes that are between 200 and 2000 kb in size (Fauron et al., 1995). Some recently sequenced plant mitochondrial genomes are bigger than this range. For example, the flowering plant *Silene conica* has a mitochondrial genome of 11.3 Mb (Sloan et al., 2012).

In the plant mitochondrial genome, there are many repeats, including huge repeats (>1 kb), tiny repeats (<100 nt), and tandem repeats.Most changes in the structure of mitochondrial DNA are thought to be caused by repeat-mediated duplication (Maréchal and Brisson, 2010). Even though plant mitochondrial genomes are usually big, their gene pools (24 core genes and 17 variant genes) are generally small. This is because, during the evolution of angiosperms, many genes were lost or moved to the nucleus, but the coding sequences of the remaining genes are very stable (Adams et al., 2002).

As a result, the mitochondrial genome can be used to create molecular markers and figure out how the mitochondrial genome evolved (Zubaer et al., 2018). When comparing the mitochondrial genomes of closely related species, new things can be learned about how evolution works and how mitochondrial genomes change over time. It can also help with the classification of species (Zubaer et al., 2018).

With the Illumina Hiseq Platform, the entire DNA of plant organelles can be sequenced. But, Illumina read lengths frequently do not span larger repetitions, which leads to an inadequate assembly of these areas and, as a result, affects both the size and the content of the genome. Third-generation sequencing approaches, which include long-read Oxford Nanopore and PacBio sequencing, can enhance the coverage and assembly accuracy of unassembled genomic areas. This is a useful tool for understanding the information contained in the plant organelle genome (Shearman et al., 2016).

As new technologies come out, more and more organelle genomic data is being made available and analyzed, and gene transfer between chloroplasts and mitochondria is thought to be a part of long-term evolution (Nguyen et al., 2020). In the past, scientists have focused on gene transfer in the DNA of organelles in angiosperms (Park et al., 2014). Because of this, more research needs to be done on the mitochondrial and chloroplast genomic information of *Theaceae* species.

*Camellia sinensis* var. *Assamica* cv.*Duntsa(C.duntsa)*, also known as “Cheng Bu Dong Cha “in Chinese. It is a tea plant native to the Chengbu Miao Autonomous County, Hunan Province, China (**Supplementary Figure 1**), with a cultivation history of over 200 years (Xinghui et al., 1998).

In botanical morphology, they are primarily small trees with large soft leaves, uplifted and glossy leaves, fuzz buds and leaves, and unique fragrances, which have the value of variety breeding and promotion (Guoben, 1987; Xinghui et al., 1998).

*C.duntsa* is often called “the Hunan province local characteristics tea plant resources”.The primary and secondary metabolites found in the fresh leaves of *C.duntsa*, such as polyphenols, amino acids, soluble sugars, and volatile organic compounds (Graham, 1992; Zhang et al., 2019b), have been linked to a lower risk of diabetes and several cancers (McCarty et al., 2020).

Nevertheless, there are currently no reports of any *C. duntsa* mitochondrial genomes. The NCBI Genbank database only has three mitochondrial genomes of tea plant species. Because of this, more research needs to be done on the mitochondrial genomic information of the *C. duntsa* species to understand how it evolved and how to classify it as a species.

Herein, the full mitochondrial genome of *C.duntsa* were identified using a combination of third-generation sequencing and second-generation sequencing techniques. After assembling and annotating the entire mitochondrial genome of *C.duntsa*, we performed an analysis of the content, organization, and phylogenetic tree of the genome. We carried out a comparative investigation of the mitochondrial of tea plant species in order to locate areas of the genome that vary, are conserved, and have been rearranged. In addition to this, we investigated the movement of genes between the chloroplast and mitochondrial genomes of *C. duntsa*. The information contained in a plant’s mitochondrial genome can be utilized to develop molecular markers and for genetic engineering, and it can also explain the phylogenetic and evolutionary links among plant species.

## 2 Methods and materials

### 2.1 DNA sequencing and botanical samples

C. *duntsa* was cultivated at Chengbu Miao Autonomous County, Hunan Province, China(26° 05’ 52” N, 110° 28’ 78” E). Liquid nitrogen frozen fresh leaves at 80°C. The Nanopore (2000cUV-Vis) sequencing device sequenced C. *duntsa* mitochondrial DNA from the sample. Fastp (v0.20.0, https://github.htm ) was used to filter the raw data, removing primer sequences and sequencing junctions, discarding reads with quality scores below Q5, and removing N values greater than 5.The Perl script Filling v0.2.1 tallied and filtered sequence data from three different sources.

### 2.2 Mitogenome assembly and annotation

Using the Canu assembly program (Koren et al., 2017), we combined data from the first three generations to generate contig sequences.Then, we check the sequences against the plant mitochondrial gene database using BLAST v2.6 (https://blast.ncbi.nlm.nih.gov/Blast.Cgi). The mitochondrial gene contig that was compared served as the seed sequence, and the original data were used to loop and expand the sequence, ultimately revealing its primary structure (or daughter ring).Newest version of Next Polish is 1.3.1(available at https://github.com/Nextomics/NextPolish ). Assembling is done with data from the second and third generations. Errors were addressed and final results from the assembling process were achieved by human inspection.

Methods used in mitochondrial. Annotation BLAST were used to search for and find matches to previously reported plant mitochondrial sequences, and then additional changes were done by hand for closely related species based on the encoded proteins and rRNA. The tRNA was annotated with the help of the program tRNA scanSE (Chan and Lowe, 2019)(http://lowel.ab.ucsc.edu/tRNAs.can-SE/ ).The program Open Reading Frame Finder (http://www.ncbi.nlm.nih.gov/gorf/gorf.html) is utilized in the process of annotating Open Reading Frames (ORF). For the assembly of the mitochondrial genome, the program OrganellarGenomeDRAW was utilized (https://chlor.obox.mpimp-golm.mpg.de/OGDraw.html).

### 2.3 Analysis of repeat structures and SSRs

To determine whether sequences are duplicates, we use the DupFinder Perl script in conjunction with the vmatch v2.3.0 software (http://www.vmatch.de/). Forward, backward, reverse, and complementary sequences of at least 30 bp in length are all recognized. For the purpose of doing simple repeat sequence analysis, we made use of MISA (https://webblast.ipk-gatersleben.de/misa/ ), a web-based tool. In this research, we found a total of six instances of one-base, two-base, three-base, four-base, five-base, and six-base repeats. SSRs have a minimum separation of 100 bp. The Tandem Repeats Finder v4.09 software (http://tandem.bu.edu/trf/trf.submit.options.html) was utilized in order to recognize tandem repeats with lengths of more than six base pairs and more than ninety-five percent of matched repeats.

### 2.4 Chloroplast-to-mitochondrial DNA transformation and RNA editing

We extracted *C.duntsa*’s chloroplast genome sequence from the NCBI Organelle Genome Resource database (OL450397). Utilizing BLAST v2.10.1, we were able to identify comparable sequences in the mitochondrial and chloroplast genomes. Define the selection criteria that will result in a successful pairing rate of seventy percent. The editing sites of the mitochondrial RNA were located by a process that involved comparing the mitochondrial RNA of *C. duntsa* to the protein that was encoded by the mitochondrial gene. The Plant Predictive RNA Editor (PREP) tool (http://prep.unl.edu/) was utilized in order to do an analysis on the data collected for this investigation.

### 2.5 Ka/Ks evaluation and phylogenetic tree building

We determined the frequencies of non-synonymous (Ka) and synonymous (Ks) substitution for each PCG between C.*duntsa* and four other plant species: *C. sinensis* (NC 043914), *C. sinensis* (OM809792), *A. thaliana* (JF729201), and *G. biloba* (KM672373).The PCG sequence was aligned using Mafft version 7.427, and the Ka/Ks analysis was performed using the MLWL-based Ka/Ks Calculator version 2.0 (Wang et al., 2010).

Utilizing MAFFT, the mitochondrial genomes of nineteen distinct species that belong to a variety of families were compared and aligned. After the sequences were aligned end-to-end, trimming was carried out using trimAl (v1.4.rev15) with the parameter -gt 0.7, then jmodeltest-2.1.10 was used to produce a prediction on the GTR status of the model. Employing the RAxML (Stamatakis, 2014)program, the GTRGAMMA model, and a bootstrap size of one thousand in order to construct an evolutionary tree with the highest probability achievable. An iterative analytic method known as Markov Chain Monte Carlo (MCMC) was used in version 3.2.7 of MrBayes to simulate a population for a total of 1,000,000 generations, and samples were taken at intervals of 100 generations. The simulation was run for a total of 1,000,000 generations. After the first 25% of the phylogenetic tree has been burnt in,a tree is produced that is consistent the majority of the time.

### 2.6 Codon usage bias patterns analysis

The codon usage bias parameter RSCU and the Effective number of codons (ENC) were calculated using an online cloud platform (cloud.genepioneer.com.).ENC was used to analyze the effect of gene base composition on codon usage preference. These two parameters are used to describe the pattern of codon usage preference. Four mitochondrial genomes from the tea plant species, including C.*duntsa, C.sinensis*(NC043914), *C.sinensis*(OM809792), and *C. sinensis* var. *Assamica*, calculate their codon preference parameters.

### 2.7 Genomic comparison of related species

The *C. duntsa* mitochondrial genome was used as a reference and compared with three other mitochondrial genomes of the tea plant species, *C.sinensis*(NC043914), *C.sinensis*(OM809792), and *C. sinensis* var. *Assamica.* The mitochondrial genomes of *C. duntsa* and *C. sinensis* (OM809792) were analyzed for covariance using Dottup software. Whole-gene covariance comparisons were performed using LASTZ version 1.02.00 to detect covariance regions.The parameters were set as follows: step = 20 and seed pattern = 12 of 19.

## 3 Results

### 3.1 Mitochondrial gene organization and features

The *C.duntsa* genomic sequence generated in this study is now archived in GenBank (accession number OL989850). In this study, the average read coverage of the assembled mitochondrial genome was more significant than 268×. Whole mitochondrial genome sequencing delivered 26,855,210 clean reads from the second-generation sequencing platform, whereas the third-generation sequencing platform generated 1,034,825 clear reads (SRA accession number SRR17287593). The sequencing and assembly statistics are shown in **Table Supplemental 1**.

**Figure 1:**
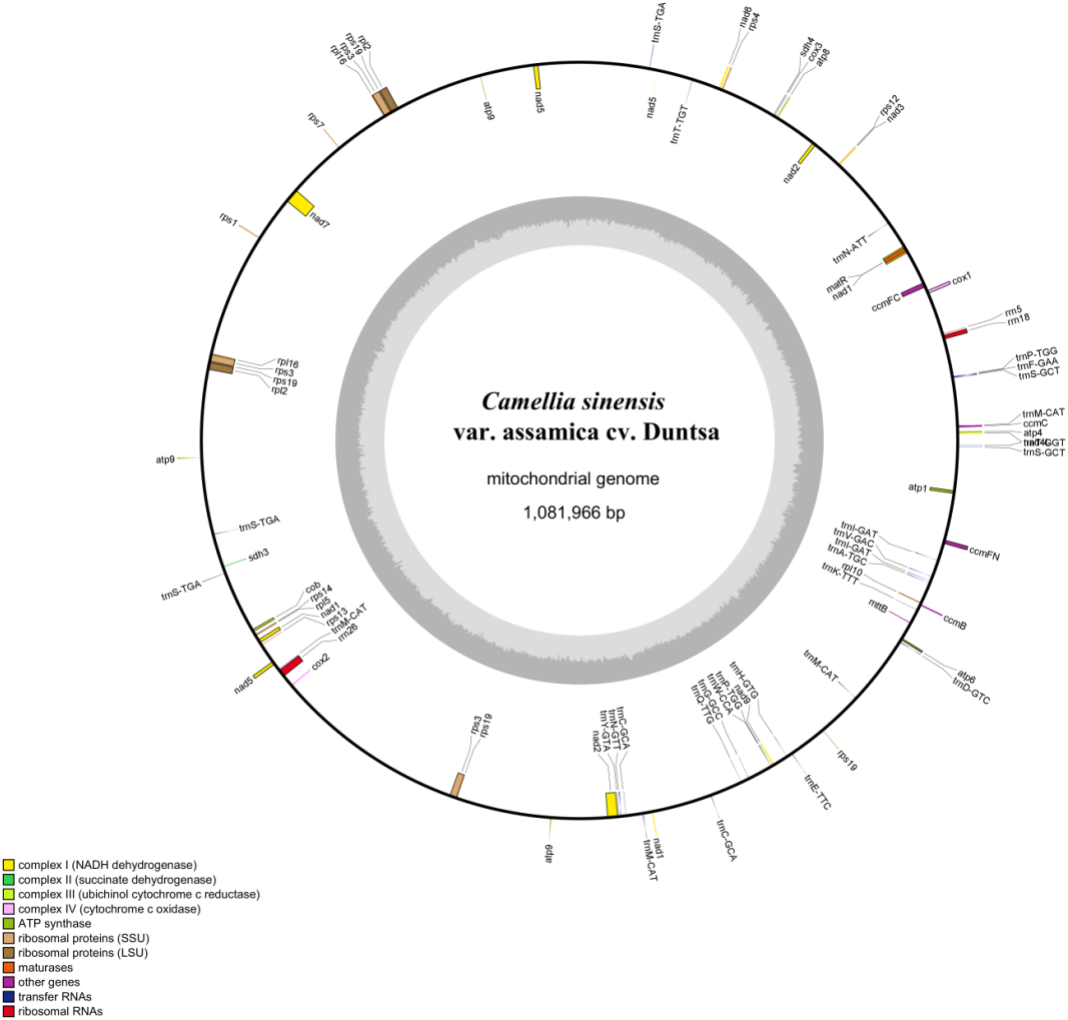
C*.duntsa* mitochondrial genome circular map.

The mitochondrial genome of *C.duntsa* consists of 1,081,996bp of closed-loop DNA molecules (**Figure 1**). *C.duntsa* mitochondrial has a nucleotide makeup of 27.03% A, 27.35% T, 22.78% G, 22.84% C, and a GC content of 45.62%. PCGs, tRNAs, and rRNAs made up 3.62%, 0.21%, and 0.52% of the total mitochondrial genome, respectively. The mitochondrial genome of *C.duntsa* has eighty-one annotated genes, including forty-seven PCGs, thirty tRNAs, three rRNAs, and one pseudogene (**Table 1**).

**Table 1:** Gene profile and organization of the *C.duntsa* mitochondrial genome.

Group of genes	Gene name
ATP synthase	atp1 atp4 atp6 atp8 atp9(3)
Cytohrome c biogenesis	ccmB ccmC ccmFC* ccmFN
Ubichinol cytochrome c reductase	cob
Cytochrome c oxidase	cox1 cox2 cox3
Maturases	matR
Transport membrance protein	mitochondrialtB
NADH dehydrogenase	nad1**** nad2**** nad3 nad4*** nad4L nad5**** nad6 nad7**** nad9
Ribosomal proteins (LSU)	rpl10 rpl16(2) rpl2**(2) rpl5
Ribosomal proteins (SSU)	rps1 rps12 rps13 rps14 rps19(4) rps3*(3) rps4 rps7
Succinate dehydrogenase	sdh3 sdh4
Ribosomal RNAs	#rrn18 rrn18 rrn26 rrn5
Transfer RNAs	trnA-TGC* trnC-GCA(2) trnD-GTC trnE-TTC trnF-GAA trnG-GCC trnH-GTG trnI-GAT*(2) trnK-TTT trnM-CAT(4) trnN-ATT trnN-GTT trnP-TGG(2) trnQ-TTG trnS-GCT(2) trnS-TGA trnS-TGA*(2) trnT-GGT* trnT-TGT* trnV-GAC trnW-CCA trnY-GTA

*:intron number; #Gene: Pseudo gene; Gene(2): Number of copies of multi-copy genes.

* for introns, the number of * for the number of introns.For example, * * * represents the number of three introns.

Most terrestrial plants contain three rRNA genes (Cavalier-tsmith, 1975; Archibald, 2011). The *rrn26* (3595 bp), *rrn18*(1923 bp), and *rrn5*(121bp), were annotated within the C.*duntsa*. In addition, the number of introns contained in the mitochondrial genomes of terrestrial plants might vary considerably. Eighteen of the annotated genes in *C.duntsa* contained introns, and eleven of those genes (*CCMFc*,*rps3*(3), *trnA-TGC*, *trnI-GAT*(2), *trnS-TGA*(2), *trnT-GGT*, and *trnT-TGT*) included an intron (**Table 1**).

### 3.2 PCGs codon use analysis

**Figure 2:**
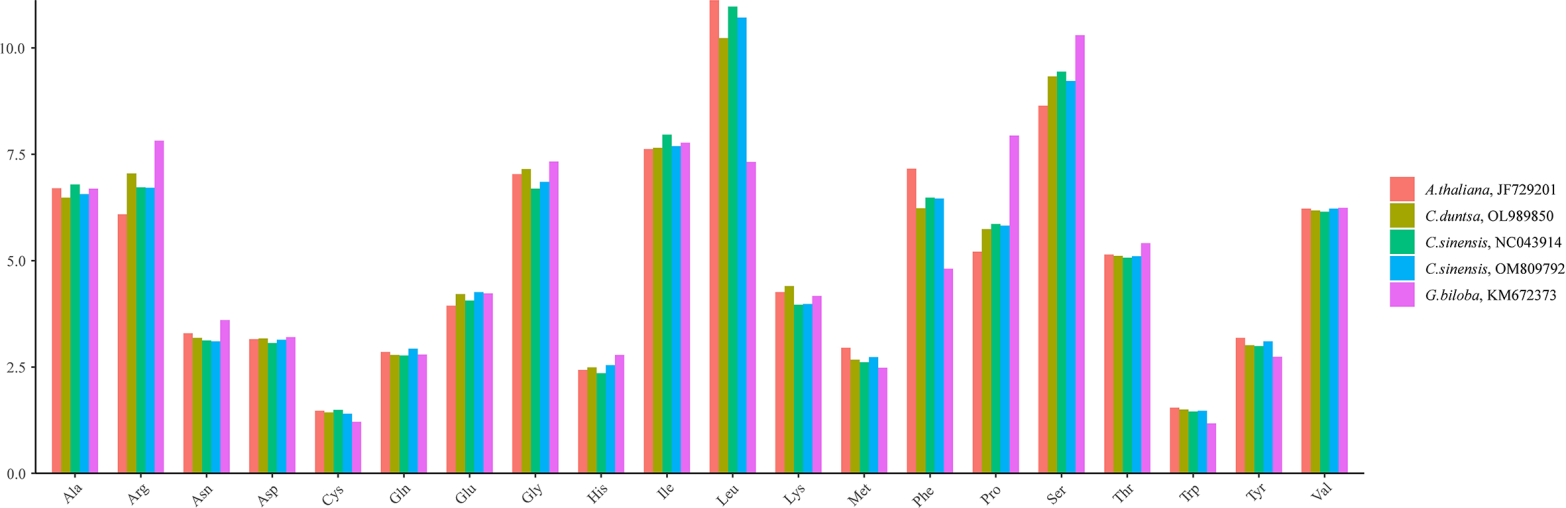
C*.duntsa* mitochondrial codon usage pattern compared to *C.sinensis*(NC043914), *C.sinensis*(OM809792), *A.thaliana*(JF729201),and *G.biloba*(KM672373).

*C. duntsa* PCGs have a length of 39,126 bp. All PCGs used ATG as the starting codon, while the terminating codons TAG, TGA, and TAA were utilized at 19.15%, 34.04%, and 46.81%, respectively (**Supplementary Table 2**). Leucine (Leu) (7.32–11.12%), Serine (Ser) (8.64–10.30%), and Aspartic Acid (Arg) (6.09–7.82%) were found to be the most frequently occurring amino acids compared to other species (**Figure 2**). In contrast, Cysteine (Cys) (1.21–1.49%) and Tryptophan (Trp) (1.17–1.54%) were identified only frequently. The Relative Synonymous Codon Usage (RSCU) of the *C. duntsa* mitochondrial genome was another aspect that we investigated. To do this, we analyzed 10,237 codons out of 47 PCGs(excluding terminated codons). 6,469 codons (63.02%) had RSCU values over 1.0, showing that they were used more than synonymous codons(**Supplementary Table 3**).

**Figure 3:**
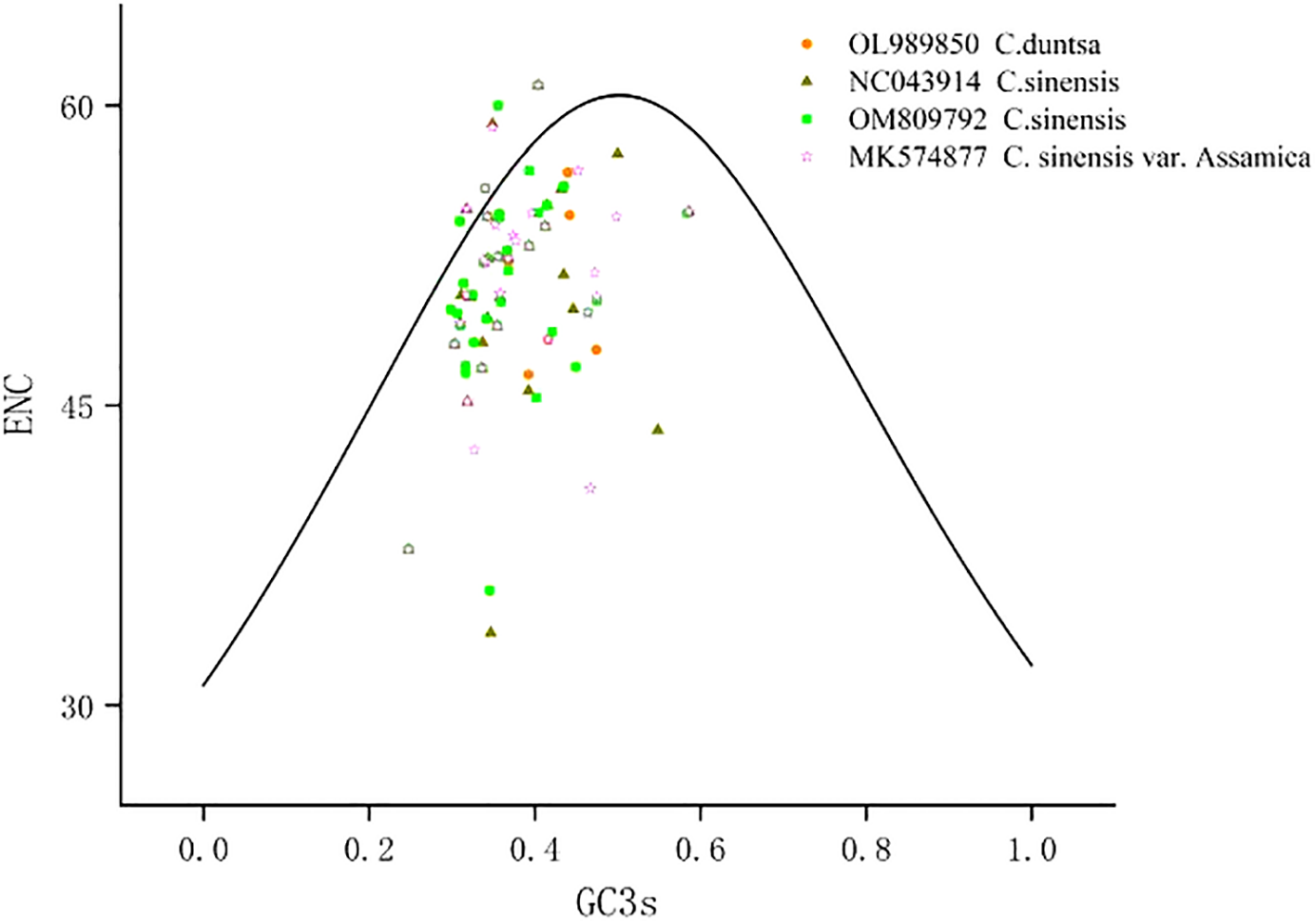
ENC plot of four *Theaceae* mitochondrial genomes.Standard curve line is calculated as follows: ENC = 2 + GC3 + 29/(GC3^2^ + (1-GC3)^2^).

According to **Supplemental Table 3**, which displays the codon usage frequency of four mitochondrial genomes from tea plant species, these four species contain between 4631 and 6351 codons with an RSCU greater than 1. The most prevalent codons among these four species were GCU (Ala), UAU (Tyr), and CAU (His) (RSCU > 1.5), whereas CAC (His) was the least common (RSCU< 0.5). The number of genes with ENC values< 35 in the mitochondrial genomes of *C.duntsa*, *C. sinensis* (NC043914), *C. sinensis* (OM809792), and *C. sinensis* var. *Assamica* is 0, 1 (*atp9*), 0 and 0 correspondingly (**Supplementary Table 4**). Four genes, including *atp4*, *rps12*, *rps7*, and *nad9*, are positioned on or above the standard curve line, while the remaining genes are located below the standard curve line (**Figure 3** and **Table 2**).

**Table 2:** Plot of ENC of genes in 4 Theaceae species mitochondrial genome.

Species	Genbank	ENC plot analysis			
		ENC values of gene on or above standard curve line		ENC values of gene below standard curve line	
		Gene number	Gene list	Gene number	Gene list
C.duntsa	OL989850	4	atp4,rps12,rps7,nad9	43	nad2,nad4L,ccmC,cox1,ccmFC,nad1,matR, nad3,atp8,cox3,sdh4,rps4,nad6,nad5, atp9,rpl2,rps19,rps3,rpl16,nad7,rps1, rpl16,rps3,rps19,rpl2,atp9,sdh3,cob, rps14,rpl5,rps13,cox2,rps3,rps19,atp9, rps19,nad4,atp6,mitochondrialtB,ccmB,rpl10, ccmFN,atp1
C.sinensis	NC043914	3	atp4,rps12,rps7	29	nad5,rps13,atp9,nad6,nad1,rps13,cox2, rps16,nad2,nad3,rps1,matR,ccmFn,ccmC, nad4,latp1,rpl10,ccmB,mitochondrialtB,atp6,nad4, orf115b,ccmF,csdh3,orf100,rpl5,rps14, cob,rps16
C.sinensis	OM809792	4	atp4,rps12,rps7,nad9	38	nad3,nad2,atp8,cox3,sdh4,rps4,nad6, nad5,nad1,matR,ccmC,nad4L,atp8,rps1, nad7,cox2,rps13,rpl5,rps14,cob,rps19, atp1,nad4,atp6,mitochondrialtB,ccmB,rpl10,ccmFN, cox2,sdh3,atp9,rps19,rpl16,ccmFC,cox1, ccmC,atp4,nad4L
C. sinensis var. Assamica	MK574877	4	atp4,rps12,rps7,nad9	28	matR,ccmFn,nad4,atp6,mitochondrialtB,ccmB,rpl10, atp1,sdh3,rpl16,rps3,rps19,rpl2,atp8, cox3,sdh4,cox1,nad7,nad4L,ccmC,cox2, rps13,rps4,nad6,atp9,nad2,nad3,rps1

### 3.3 Analysis of synonymous and nonsynonymous replacement rates

**Figure 4:**
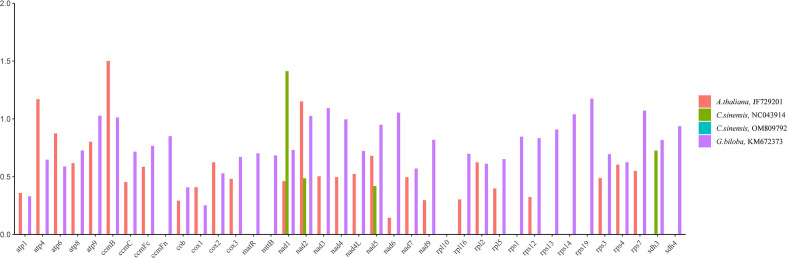
Ka/Ks ratios of thirty-eight PCGs in *C.duntsa*,*C.sinensis*(NC043914),*C.sinensis*(OM809792), *A.thaliana*(JF729201),and *G.biloba*(KM672373).

Calculating non-synonymous substitutions (Ka) and synonymous substitutions (Ks) is essential for reconstructing phylogenies and studying the evolution of protein-coding sequences among closely related species. Positive selection (Ka/Ks >1), neutral selection (Ka/Ks =1), and negative selection (Ka/Ks<1) are all possible outcomes. Here, we analyzed the Ka, Ks ratio of thirty-eight PCGs present in the mitochondrial genomes of *C. duntsa*, *C. sinensis* (NC043914), *C. sinensis* (OM809792), *A. thaliana* (JF729201), and *G. biloba* (KM672340) (**Figure 4**). Ka/Ks values for the thirty-eight shared PCGs between *C. duntsa* and *C. sinensis* (OM809792) were zero. For the thirty-five shared PCGs between *C. duntsa* and *C. sinensis* (NC043914), the Ka/Ks values were also zero. In contrast, eleven of the thirty-eight genes shared by all four species have Ka/Ks values that are larger than 1, indicating that they were positively chosen during the process of evolution. These genes include *nad2*, *ccmB*, *atp4*, *nad6*, *atp9*, *rps7*, *rps19*, *rps14*, *ccmB*, *nad3* and *nad1*.

### 3.4 Repeat analysis of the *Camellia sinensis* var. *Assamica* cv. *duntsa* mitochondrial genome

**Figure 5:**
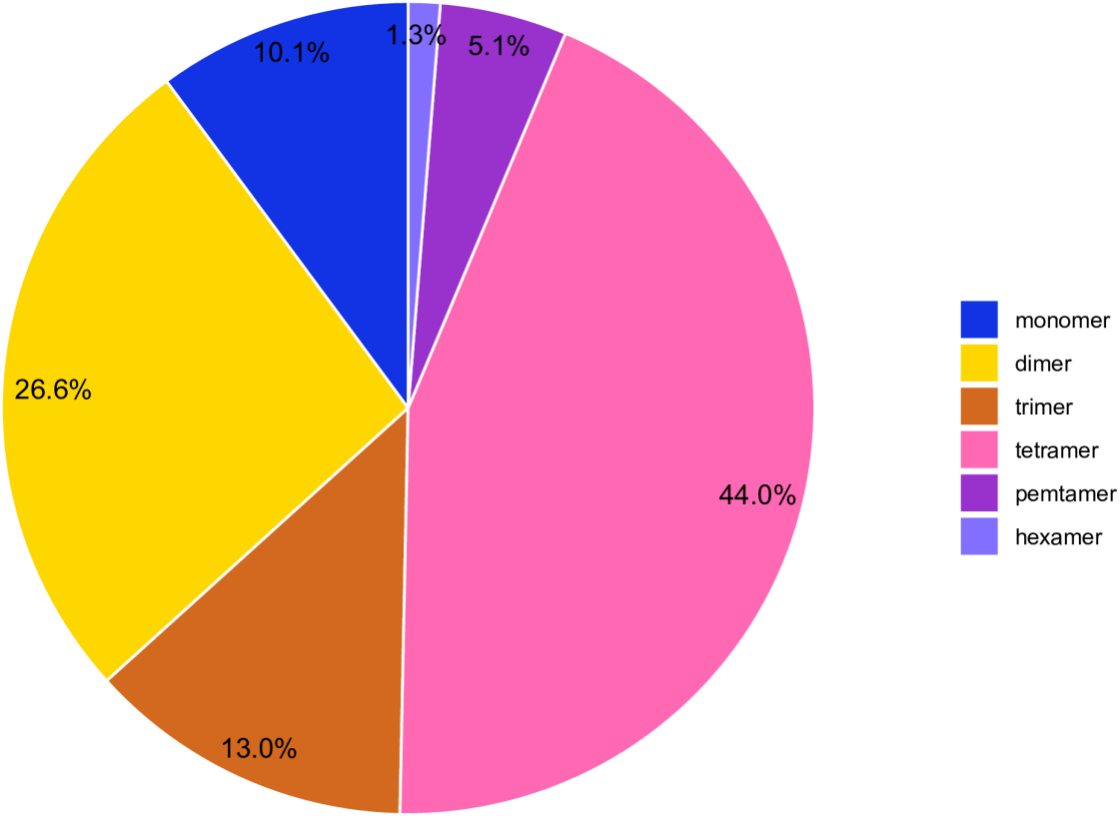
C*.duntsa* SSR distribution. Different hues denote SSRs. The graphic shows SSR percentages.

Simple sequence repeats (SSRs) are sequence-repeating units ranging in length from one to six base pairs (Qiu et al., 2010).SSRs are useful due to their polymorphism, ease of PCR detection, codominant inheritance, and extensive genome coverage (Powell et al., 1996).The *C.duntsa* mitochondrial genome SSRs were identified using the Tandem Repeats Finder program. There were 316 SSRs discovered, and **Figure 5** displays the proportion of each kind. According to weighted SSRs, tetramer repeats comprised 43.90% of the total, followed by dimer repeats at 26.00%. AGAA repetitions were the most common form of tetramer SSRs, accounting for 7.10%, whereas CT repeats were the most common form of dimer SSRs, accounting for 17.80%. The *C.duntsa* mitochondrial genome has only four hexamers SSRs, located between *atp9* and *rpl2*, *rpl2-2* and *atp9-2*, *cox2* and *rps3-3*, and *rps19-3* and *atp9-3*. In **Supplemental Table 5**, the positions of the pentamer and hexamer are displayed.

**Figure 6:**
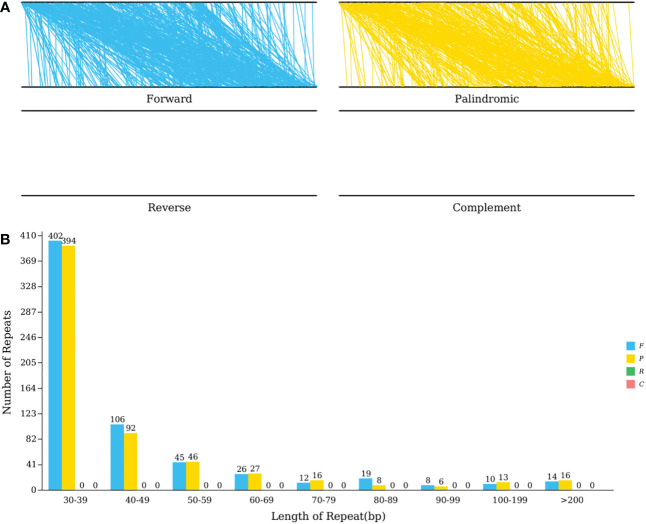
C*.duntsa* mitochondrial repeats. **(A)** The mitochondrial genome and its forward copy both contain forward repeats.Palindromic repeats in mitochondrial genome and its reverse complement. **(B)** *C.duntsa* mitochondrial genome repeat length distribution. Histograms display the repeat number of given lengths.

Tandem repetitions refer to the approximately 1-200 base repeat units repeated repeatedly. They are widespread in eukaryotic and certain prokaryotic genomes (Huan and Jie, 2005). The mitochondrial genome of *C. duntsa* has thirteen sequence repetitions with a significance level greater than 95%, ranging in length from 12 to 39 bp, according to **Supplemental Table 6**. The REPuter program (Kurtz et al., 2001) identified the *C. duntsa* mitochondrial genome’s interspersed repeat sequence. The longest forward repetition was 92,293bp while the longest palindromic repeat was 88,112bp. Both types of repeats are mostly made up of 30–39 bp repeats (**Figure 6**).

### 3.5 The identification of potential RNA editing sites in PCGs

**Figure 7:**
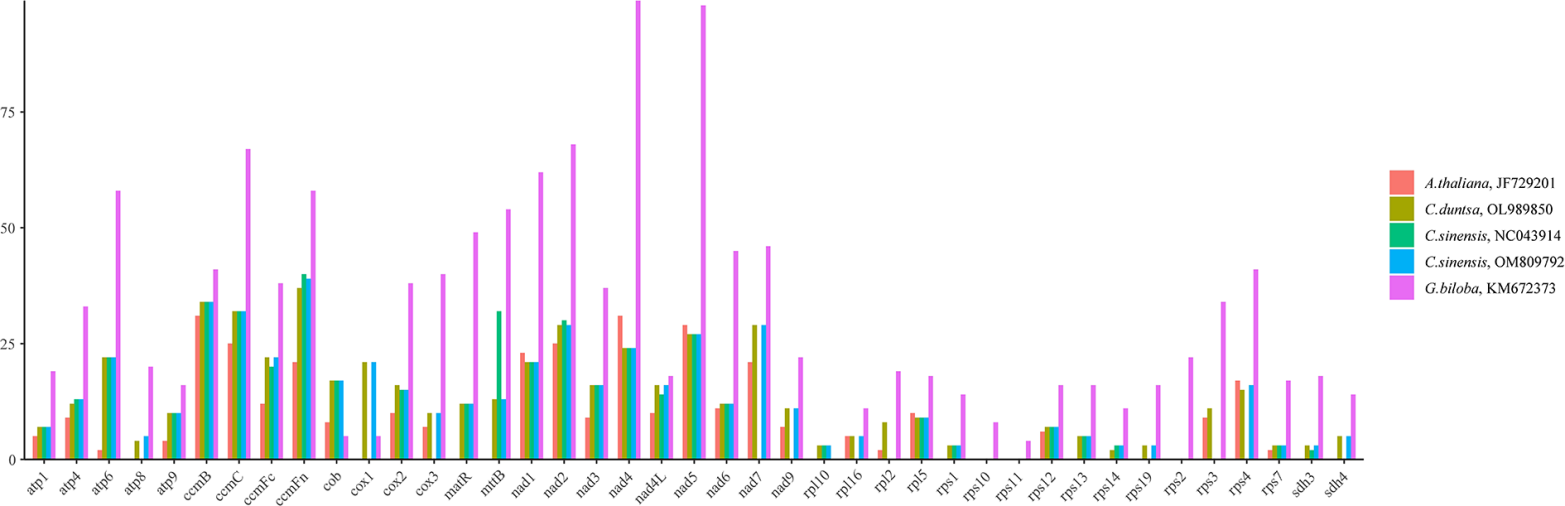
The distribution of RNA editing sites across the PCGs of the mitochondrial genomes of five different angiosperms.

RNA editing, the insertion, deletion, or replacement of nucleotides in a transcribed RNA’s coding region, is widespread in land plants other than mosses. (Malek et al., 1996; Brennicke et al., 1999).This work predicted the RNA editing sites of forty-one PCGs conserved among five angiosperms to search for similarities. The fact that *G.biloba* (KM672373;1315) contained more RNA editing sites than *A. thaliana* (JF729201), *C. duntsa* (OL989850), *C. sinensis* (NC043914), and *C. sinensis* (OM809792) (351, 536, 436, and 522) implies that these positions are highly conserved in tea plant PCGs (**Figure 7**).

Among the RNA editing sites in *C.duntsa* PCGs, *ccmFn* contains the greatest number, 37, accounting for 6.90% of all editing sites.The *rps14* has minor editing sites, 2, accounting for 0.3%. The first position of triplet codes contained 32.07% of these 536 RNA editing sites, whereas the second position had 67.93%.(**Supplementary Table 7**).The water-repelling properties of 43.74 percent of the amino acids did not change, 7.29% became hydrophilic, and 48.22% became hydrophobic.*Atp6*, *atp9*, *cox2*, and *ccmFc* of the C.*duntsa* mitochondrial genome are prematurely terminated due to the presence of RNA editing. Our research also indicated that following RNA editing, leucine was changed to 45.61% of the edited amino acids, which suggests that leucine was the predominant amino acid among the projected editing codons(**Supplementary Table 7**).

### 3.6 Genome size and GC content in *Camellia sinensis* var. *Assamica* cv. *duntsa* and other species

**Figure 8:**
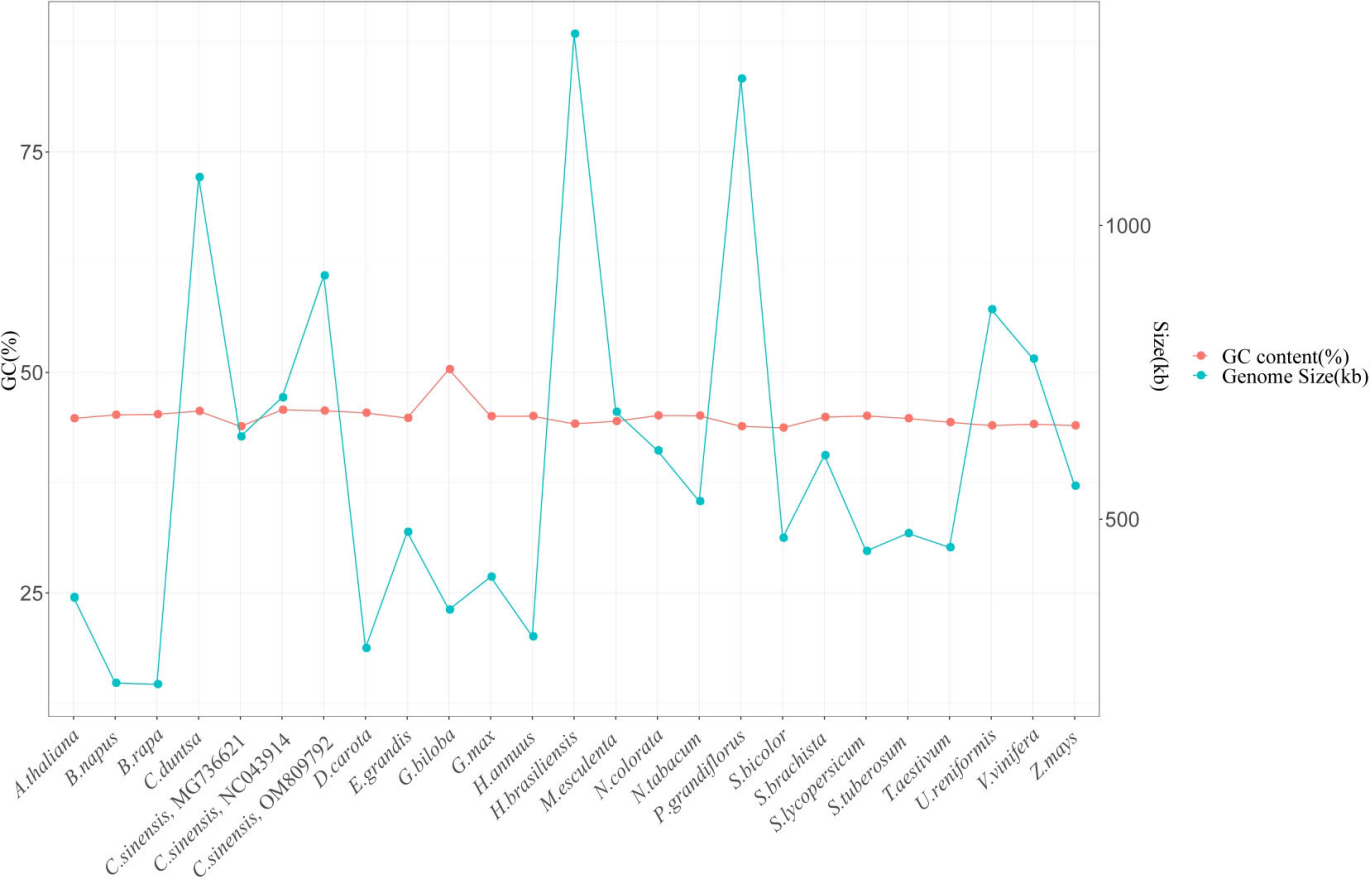
Gene content (GC) and genome size (kb) of twenty-four plant mitochondrial genomes.

It is generally accepted that the size of the genome and the proportion of GCs it contains are two of the most critical factors determining the genomes of plant organelles. The mitochondrial genomes of twenty-three other plant species were analyzed, and their sizes and percentages of GC content were compared to the *C. duntsa* mitochondrial genome. Of the twenty-three species, one was a gymnosperm, two were monocots, and the remaining twenty were dicotyledons. **Supplementary Table 8** lists these plants’ species abbreviations and mitochondrial genome access numbers. There was a wide range in plant mitochondrial genome size, from 219,736 Kb (*Brassica rapa*) to 1,325,823 Kb (*Hevea brasiliensis*). Although there is a wide range in plant mitochondrial genome size, the GC content (often approximately 44%) shows that the mitochondrial genome has remained generally stable throughout evolution (**Figure 8**).

### 3.7 Mitochondrial genome comparison in tea plant species

**Figure 9:**
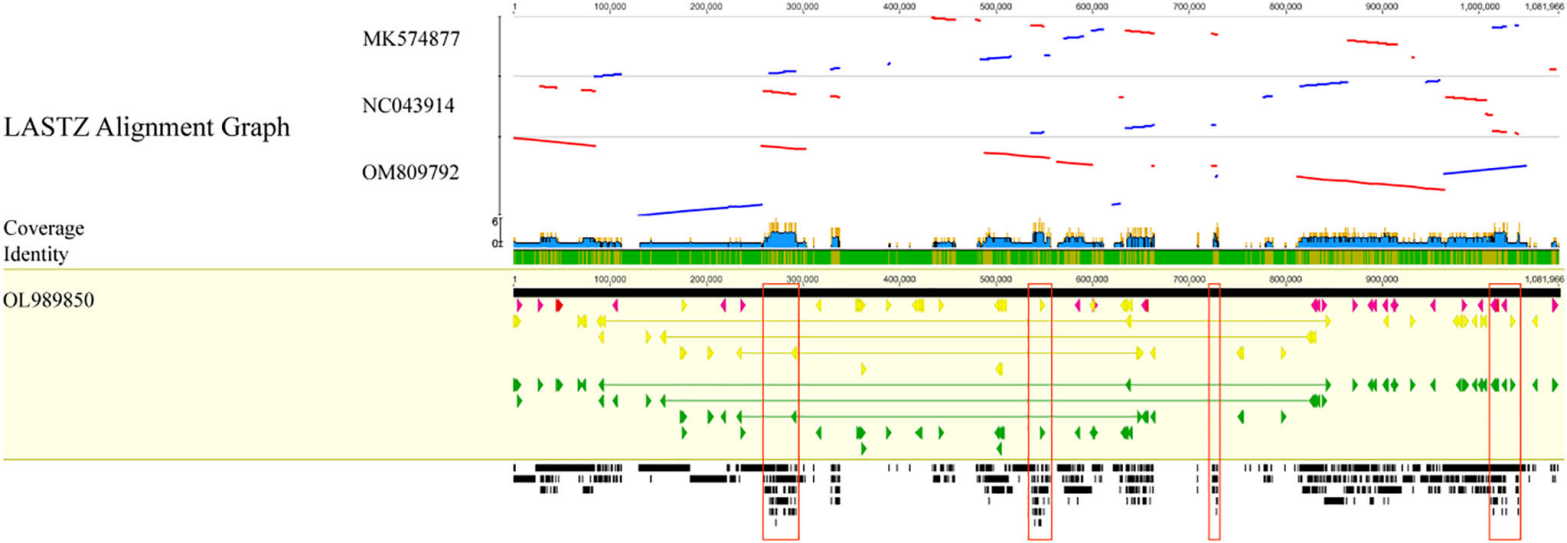
Four *Theaceae* mitochondrial genomes aligned collinearly.High-scoring segment co-direction pairings are shown by blue bars, and reversed pairs are shown by red bars. Arrows in yellow indicate coding sequences, red ribosomal RNA genes, purple transfer RNA genes, green protein-coding genes, and gray exonic areas. In this case, the larger the black squares, the more genetic material the two species share.Conservative regions are red. These sections comprise nad5, atp9, cox 2, rps 3, trnA-TGC, trnI-GAT, rrn18, trnV-GAC, and ccmFN.

As displayed in **Figure 9**, the *C. duntsa* mitochondrial genome served as a standard for evaluating the outcomes of a whole-genome correlation match performed on four different tea plant taxa. In the gene region of the mitochondrial genomes of these related species, there was a higher level of similarity with *C. duntsa*; the most conserved genes were *nad5*,*atp9*,*cox2*,*rps3*,*trnA-TGC*,*trnI-GAT*, *rrn18*, *trnV-GAC*, and *ccmFN*. Compared to those of the other two tea plant species, the mitochondrial genomes of *C. duntsa* and *C. sinensis* (OM809792) were more similar to one another. In addition, the arrangement of the mitochondrial genomes of C. *duntsa* and *C. sinensis* (OM809792) shown that substantial variation still existed (**Supplementary Figure 3**).

**Figure 10:**
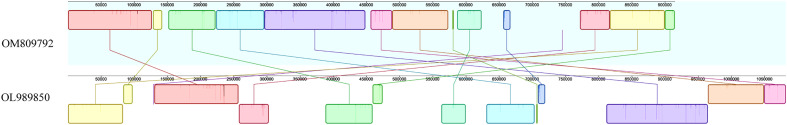
Mauve comparison between *C.duntsa* and *C.sinensis* genomes (OM809792).

In order to evaluate mitochondrial genome rearrangement and collinearity, the mitochondrial genome of *C. duntsa* and *C.sinensis* (OM809792) were compared using the Mauve and Lastz programs.C. *duntsa* and *C.sinensis* mitochondrial genome (OM809792) were split into fourteen locally collinear blocks (LCBs). Both the size of these LCBs and their position in relation to one another varied greatly between the two tea plant species that were researched (**Figure 10**). In addition, the majority of LCBs contained gene sequences, with a total of thirty-one genes involved.The examination of the rearrangement revealed that the mitochondrial genomes of *C. duntsa* and *C. sinensis*(OM809792)had undergone a significant amount of rearrangement (**Supplementary Table 9**).

### 3.8 Tea plant species mitochondrial genome duplication and loss

With sequencing technology advancing rapidly, the entire plant mitochondrial genome has been constructed and reported, enabling comparative research of mitochondrial genome features across plant species (Wei et al., 2016). Four mitochondrial genomes from this family can now be accessed: *C.duntsa*, *C.sinensis*(NC043914), *C.sinensis(*OM809792), and *C. sinensis* var. *Assamica*. These four mitochondrial genomes each include a total of 47, 30, 42, and 44 protein-coding genes, respectively(**Supplementary Table 10**).

In distinct species, gene duplication and gene loss have been documented. For instance,*rpl16*, *rpl2*, *atp9*, *rps3*, and *rrn18* were replicated in the C.*duntsa* genome, *rps13* and *rps16* replicated in the *C.sinensis* (NC043914) genome, *atp4*, *atp8*, *ccmC*, *cox2*, and *nad4L* replicated in the *C.sinensis* (OM809792) genome, and atp9, nad1, nad2, nad9, rps19, sdh3 replicated in the *C. sinensis* var. *Assamica.*

*C.duntsa* and *C. sinensis* var. *Assamica* has the complete mitochondrial genome, with only one gene (*rps16*) being lost, *rpl2*, *rps16*, and *rps3* from *C.sinensis* (OM809792) being lost, and *C.sinensis* (NC043914) losing most of eleven genes(**Supplementary Table 10**).

### 3.9 Phylogenetic analysis

**Figure 11:**
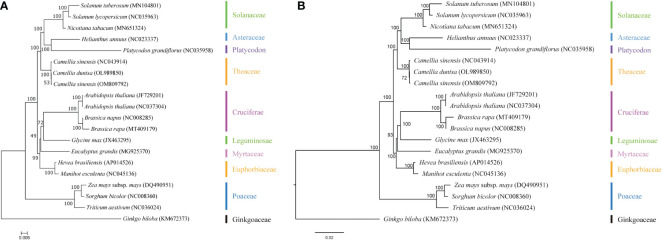
C*.duntsa*’s nineteen plant phylogenetic connections. Outgroup: *Triticum aestivum*, *Sorghum bicolor*, *Ginkgo biloba*, *Zea mays*. **(A)** Maximum-likelihood phylogenies. **(B)** Bayesian phylogeny.

To better understand the evolution of the *C. duntsa* genome, a taxonomic study was carried out on the mitochondrial genome of the *C. duntsa* species, in addition to nineteen other published plant mitochondrial genomes.The phylogenetic trees were constructed through a combination of the maximum likelihood method and the Bayesian method. The species that were taken into consideration are listed, together with their respective NCBI accession numbers, in **Supplemental Table 11**.In the study of phylogenetic relationships,*Sorghum bicolor*, *Triticum aestivum*, *Zea mays*, and *Ginkgo biloba* are employed as outgroups. Both analysis approaches produce mutually consistent results for classifying the phylogenetic tree. This evolutionary tree shows that gymnosperms and dicots are distinct from monocots and dicots, respectively (**Figure 11**). The phylogenetic tree’s clustering matches these species’ family and genus affiliations, confirming mitochondrial genome-based clustering. The phylogenetic tree also demonstrated that *C.duntsa*, *C.sinensis*(NC 043914), and *C.sinensis*(OM809792) are linked. Our mitochondrial genome study provides a solid foundation for future tea plant relatedness studies.

### 3.10 Migration of chloroplast DNA in mitochondrial genome

Using BLAST v2.10.1, we performed homologous fragment analysis on C.*duntsa* mitochondrial and chloroplast genome segments with > 70% similarity (**Supplementary Figure 4**). It was determined that the C.*duntsa* mitochondrial genome sequence (1,081,966 bp) is roughly 6.9 times longer than the chloroplast genome (157,026 bp). eighteen gene-containing homologous segments were discovered between the chloroplast and mitochondrial genomes of C.*duntsa* (**Table 3**). These fragments comprised 1.56 and 10.7 percent of the mitochondrial and chloroplast genomes, respectively. The following twelve mitochondrial genes were identified: *trnV-GAC*,*trnP-TGG*,*trnW-CCA*,*trnI-GAU*,*trnA-UGC*,*trnD-GTC*,*trnN-GTT*,*trnM-CAT*,*trnI-CAU*,*rrn16*,*rrn23*,*rrn18*,and *rpl2* (**Table 3**).

**Table 3:** Plot of ENC of genes in 4 Theaceae species mitochondrialgenomes.

Sequence name	chloroplast genome		mitochondrial genome		Identity (%)
	Gene	Sequence posison (bp)	Gene	Sequence posison (bp)	
1	rps12,rrn16,rrn23,rrn4.5,trnA-UGC,trnI-GAU,trnV-GAC	100,306-109,877	trnV-GAC,rrn16,trnI-GAU,trnA-UGC,rrn23	1,020,938-1,011,367	100
2	rpl2,rpl23	155,277-156,965	rpl2	51,236-49,548	99.882
3	psbE,psbF,psbJ,psbL	66,255-67,196	/	900,981-901,958	87.271
4	petG,petL,trnP-UGG,trnW-CCA	68,176-69,226	trnP-TGG,trnW-CCA	902,317-903,324	85.741
5	ndhJ,ndhK	51,347-52,089	/	1,028,905-1,028,171	81.794
6	atpB,atpE	54,522-55,008	/	1,055,976-1,056,456	86.895
7	rpoB	25,907-26,296	/	895,392-895,799	81.971
8	rrn16	139,493-140,356	/	47,662-46,804	74.045
9	psbC	36,657-36,803	/	843,420-843,566	95.918
10	trnD-GUC	31,892-32,040	trnD-GTC	985,399-985,251	91.275
11	rpl16	84,568-84,744	/	165,181-165,347	87.079
12	ycf2	94,245-94,370	/	666,069-665,950	88.889
13	trnN-GUU	132,310-132,392	trnN-GTT	830,649-830,566	96.429
14	trnI-CAU	88,651-88,726	trnI-CAU	6,689-6,614	97.368
15	trnM-CAU	54,208-54,284	trnM-CAT	840,336-840,412	93.506
16	ndhA	122,869-122,910	/	1,020,307-1,020,266	100
17	ycf2	150,687-150,739	/	1,048,980-1,048,928	90.566
18	trnD-GUC	31,998-32,029	/	580,871-580,840	96.875

## 4 Discussion

### 4.1 Mitochondrial genome structure and size variations

Mitochondria give plants the energy they need to live. Plant mitochondrial genomes are more complicated than animal ones because they are bigger and have more repeated sequences (Kozik et al., 2019). The key features of the Chinese *C.duntsa* mitochondrial genome are described in this article. Black tea made from its tender buds is a rare resource with a rich fragrance, mellow taste, and convergence. Many mitochondrial genomes are circular, but some are linear, like those found in Polytomella parva (Zhang et al., 2018). The circular *C.duntsa* mitochondrial genome that was published here is 1,081,966 bp long, and it has a GC content of 45.62%. The GC content was comparable to that of other sequenced plant mitochondrial genomes, for example, *B. chinense*, 45.68% (Qiao et al., 2022);*B. juncea*, 45.24% (Chang et al., 2011).

The mitochondrial genome contains many types of repeats, including tandem, short, and large repeats (Gualberto et al., 2014; Guo et al., 2017). Repeated sequences have been shown to be crucial to intermolecular recombination in previous studies of mitochondria. As a result, repetitive sequences play a crucial role in the assembly of the mitochondrial genome (Dong et al., 2018). SSRs, longer tandem repeats, and non-tandem repeats were the primary targets of this study (**Figure 5**-**6**). Numerous repetitive sequences have been discovered in the mitochondrial genome of *C.duntsa*, which may be evidence of frequent intermolecular recombination that has dynamically altered the structure and conformation of the mitochondrial genome over the course of evolution. We also compared the genome of *C.duntsa* to those of other terrestrial plants to learn more about its structure and organization. In conclusion, *C.duntsa*’s mitochondrial genome shares features that are common among other land-dwelling green plants.

Mitochondrial genomes for the four tea plant species ranged in size from 701,719 to 1,081,966bp. From the shape and size of the mitochondrial genome in tea plant species, it might be possible to figure out phylogenetic relationships and the rate at which species change.The size and structure of the mitochondrial genome are very similar in closely related species,such as *R. simsii* and *R. pulchrum* (Xu et al., 2021),the mitochondrial genome may be an important tool for understanding plant evolution and defining taxonomic taxonomy.

*C.duntsa* has forty-seven PCGs, thirty tRNAs, three rRNAs, and one pseudogene in its mitochondrial genome, it is the tea plant species with the longest genome that has been identified to this point.In the mitochondrial genomes of four distinct species of tea plant, researchers discovered several copies of the same gene.For example, genome *atp9* has three copies in the mitochondrial genome of *C.duntsa* and two copies in the mitochondrial genome of *C. sinensis* var. *Assamica*. In addition, the mitochondrial genome of the *C.duntsa* species had a greater number of annotated genes than the mitochondrial genome of the *C.sinensis* (NC043914) species. These genes included *atp8*, *cox1*, *cox3*, *nad7*, *nad9*, *rpl16*, *rpl2*, *rps19*, *rps3*, *rps4*, and *sdh4*.Therefore, plant mitochondrial genomes are vital for future research, as they will reveal information on the evolution of genomes that has not been discovered before.

### 4.2 Phylogenetic and Mitochondrial genome comparison

The results of the mitochondrial phylogenetic trees of three tea plant species and seventeen other plant species were coherent with the taxonomic information linked with those species. These findings illustrate the possibility of employing information acquired from organelle genomes in plant phylogeny studies. (Wu and Ge, 2012).The authors of this study made use of classification trees to show that *C. sinensis* and *C. duntsa* are closely related to one another (**Figure 11**).

The mitochondrial genome of *C. duntsa* was used as a reference in this study. According to a whole-genome correlation match, three tea plant species share more genomic areas with *C.duntsa* than interval sections. This demonstrates that segments of the mitochondrial genome containing genes are more conserved throughout tea plant species than are segments containing intervals.

This study found nine conservative genes: *nad5*, *atp9*, *cox2*, *rps3*, *trnA-TGC*, *trnI-GAT*, *rrn18*, *trnV-GAC*, and *ccmFN*. These genes will help researchers understand more about evolution and classify tea plant species (Li et al., 2021). Fourteen LCBs were found in the mitochondrial genomes of *C.duntsa* and *C.sinensis* (OM809792). These LCBs vary greatly in size and location (**Figure 10**).

Moreover, the majority of LCBs contain DNA sequences. Many genes are rearranged between the mitochondrial genome of *C.duntsa* and *C.sinensis* (OM809792), their mitochondrial genome has extremely complicated variations that have the potential to result in hybridization and duplication.These findings are consistent with a prior study that found extensive diversity and rearrangement in the plant mitochondrial genome (Xu et al., 2021). Furthermore, the order of genes in the mitochondrial genome of plants varies widely,the structure of mitochondrial DNA is useful for tracing common ancestry among diverse species (Xiao et al., 2017).

RNA editing is a process that occurs following transcription. It occurs in the mitochondrial genomes of higher plants and aids in the folding of proteins. The study of RNA editing sites aids in the comprehension of plant mitochondrial gene expression. Arabidopsis contains 441 RNA editing sites for 36 genes (Unseld et al., 1997), while rice has 491 RNA editing sites for 34 genes(A et al., 2019), according to previous study. This investigation uncovered 536 RNA editing sites within 41 genes.After RNA editing, the water-repelling properties of 43.74 percent of the amino acids did not change, 7.29% became hydrophilic, and 48.22% became hydrophobic. There are consistent results in the mitochondrial genome of *B. chinense* (Qiao et al., 2022).

Prior research has found that editing at the second codon location accounts for almost half of all RNA editing (Cheng et al., 2021). Consistent with earlier research, 67.93% of editing sites in the *C.duntsa* mitochondrial genome were found at the second base of the triplet codon. The identification of RNA editing sites gives crucial information for predicting the function of genes containing new codons.

Ka/Ks analysis of mitochondrial genome from *C.duntsa*, *C.sinensis*(NC043914), *C.sinensis*(OM809792), *A.thaliana*, and *G.biloba* shows that the PCGs of the *C.duntsa* mitochondrial gene are generally preserved. However, we also found genes in which the Ka/Ks >1, such as nad2, ccmB, atp4, nad6, atp9, rps7, rps19, rps14, nad3, and nad1. This shows that positive selection has worked on these coding genes over the course of their evolutionary history. In studies of gene selection and evolution in the *Theaceae* family, high Ka/Ks gene ratios are very important.

Besides, we investigated the amount of GC found in the mitochondrial genomes of *C.duntsa* and other other green plants. The findings provide support for the theory that the GC content of higher plants maintains a high degree of consistency across time (Cheng et al., 2021).

### 4.3 Patterns of codon use bias and evolution

On animals and insects, phylogenetic and evolutionary analyses based on codon use bias are frequently undertaken, however less studies have been conducted on plants (Sun et al., 2009).The low ENC mitochondrial genes (ENC**<** 35; **Supplementary Tables 4**) may be prone to mutation, while the codon usage bias observed in the remaining genes(ENC>35)is most likely due to natural selection or another cause (Wright, 1990). Our research reveals that just one mitochondrial gene (atp9,ENC=33.62) in the *Theaceae* family has had its codon use bias altered by mutation, whereas the other genes’ codon usage bias was altered by natural or artificial selection. These results suggested that NEC analysis of the mitochondrial genome would be valuable for studying the evolutionary history of plants belonging to the tea plant family.

### 4.4 Gene transfer between mitochondrial and chloroplast genomes

Sequencing analyses of the nuclear, mitochondrial, and chloroplast genomes have shown that genes can move between different genomes inside a cell (Nguyen et al., 2020).There has been an upsurge in the publication and analysis of data relevant to organelle genomes as a result of the introduction of novel methods (Park et al., 2014). It is thought that plant long-term evolution includes gene transfer between chloroplast and mitochondria (Nguyen et al., 2020).There are eighteen gene-containing homologous regions that are shared between the chloroplast and mitochondrial genomes of *C.duntsa* (**Table 3**), which points to the possibility of horizontal transfer of genes among organelle genomes.From the chloroplast, nine tRNA genes were moved to the mitochondria. It is frequent in angiosperm for tRNA genes to be transferred from the genome of the chloroplast to the genome of the mitochondria (Bi et al., 2019).

Although their homologous fragments have been found, twenty-two chloroplast genes in the mitochondrial genome of *C.duntsa* remain unannotated. These include one tRNA (*trnD-GUC*), three ribosomal genes (*rrn16*, *rrn16*, and *rrn4.5*), one photosynthetic gene (*psbC*), and three NADH dehydrogenase gene (*ndhJ*, *ndhK*, and *ndhA*)(**Table 3**). The ribosome complex, which is partially generated by the ribosomal genes, is responsible for the synthesis of the proteins that are necessary for the correct operation of the cell. During the process of photosynthesis, genes that code for photosynthetic processes and genes that code for NADH dehydrogenase work together to produce the photosynthetic system II and the chloroplast NADH dehydrogenase complex, respectively (Sazanov et al., 1998).It is hypothesized that *psbC*, a photosynthesis-related gene, evolved in the mitochondria of *C.duntsa* due to the transfer of chloroplast genes to mitochondria. By studying the genomes of plant chloroplast and mitochondria, additional insights will be acquired into the evolution of phylogenetic analyses and molecular markers of these specially arranged genomes.

## 5 Conclusions

This research mapped out the complete mitochondrial genome of *C.duntsa*. Various aspects of the genome have been investigated, including its organization, genome comparisons with those of closely related species, and horizontal gene transfer between the mitochondrial and chloroplast genomes. Several tea plant plants lack the *atp8*, *cox1*, *cox3*, *nad7*, *nad9*, *rpl16*, *rpl2*, *rps19*, *rps4*, and *sdh4* genomes in their mitochondrial genomes. However, *C. duntsa* maintains the integrity of these genes. Those findings can be utilized in the development of molecular markers.

Phylogenetic relationships, patterns of biased codon usage, and evolutionary history were investigated and studied.Our research identified nine genes associated with conservatism: *nad5*, *atp9*, *cox2*, *rps3*, *trnA-TGC*, *trnI-GAT*, *rrn18*, *trnV-GAC*, and *ccmFN*. These genes will aid scientists in understanding evolution and classifying tea plant species.

The complexity of the organelle genome variation among the tea plant species is demonstrated by this study. The findings would be helpful in understanding the mechanisms of evolution as well as the taxonomy of species within the *Camellia* family.

## Data availability statement

The original contributions presented in the study are included in the article/**Supplementary material**. Further inquiries can be directed to the corresponding author.

## Author contributions

JL and LX: design; validation; resources; database gathering; writing; preparation; analysis; and editing of initial drafts. HT and HL: methods. HL, NZ, and JT are all types of software. LX: finance acquisition. All authors contributed to the article and approved the submitted version.

## References

[B1] Adams K. L. Qiu Y.-L. Stoutemyer M. Palmer J. D. (2002). Punctuated evolution of mitochondrial gene content: High and variable rates of mitochondrial gene loss and transfer to the nucleus during angiosperm evolution. Proc. Natl. Acad. Sci. 99, 9905–9912. doi: 10.1073/pnas.042694899 12119382 PMC126597

[B2] Archibald J. M. (2011). Origin of eukaryotic cells: 40 years on. Symbiosis 54, 69–86. doi: 10.1007/s13199-011-0129-z

[B3] Bi C. Paterson A. H. Wang X. Xu Y. Ye N. (2019). Corrigendum to “Analysis of the complete mitochondrial genome sequence of the diploid cotton gossypium raimondii by comparative genomics approaches”. BioMed. Res. Int. 2019, 1–2. doi: 10.1155/2019/9691253 PMC6735218 31559314

[B4] Birky C. W. (1995). Uniparental inheritance of mitochondrial and chloroplast genes: mechanisms and evolution. Proc. Natl. Acad. Sci. U. S. A. 92, 11331–11338. doi: 10.1073/pnas.92.25.11331 8524780 PMC40394

[B5] Brennicke A. Marchfelder A. Binder S. (1999). RNA Editing. FEMS Microbiol. Rev. 23, 297–316. doi: 10.1111/j.1574-6976.1999.tb00401.x 10371035

[B6] Cavalier-tsmith T. (1975). The origin of nuclei and of eukaryotic cells. Nature 256, 463–468. doi: 10.1038/256463a0 808732

[B7] Chang S. Yang T. Du T. Huang Y. Chen J. Yan J. . (2011). Mitochondrial genome sequencing helps show the evolutionary mechanism of mitochondrial genome formation in brassica. BMC Genomics 12, 497. doi: 10.1186/1471-2164-12-497 21988783 PMC3204307

[B8] Chan P. P. Lowe T. M. (2019). tRNAscan-SE: Searching for tRNA genes in genomic sequences. Methods Mol. Biol. Clifton NJ 1962, 1–14. doi: 10.1007/978-1-4939-9173-0_1 PMC6768409 31020551

[B9] Cheng Y. He X. Priyadarshani S. V. G. N. Wang Y. Ye L. Shi C. . (2021). Assembly and comparative analysis of the complete mitochondrial genome of suaeda glauca. BMC Genomics 22, 167. doi: 10.1186/s12864-021-07490-9 33750312 PMC7941912

[B10] Cusimano N. Wicke S. (2016). Massive intracellular gene transfer during plastid genome reduction in nongreen orobanchaceae. New Phytol. 210, 680–693. doi: 10.1111/nph.13784 26671255

[B11] Dong S. Zhao C. Chen F. Liu Y. Zhang S. Wu H. . (2018). The complete mitochondrial genome of the early flowering plant nymphaea colorata is highly repetitive with low recombination. BMC Genomics 19, 614. doi: 10.1186/s12864-018-4991-4 30107780 PMC6092842

[B12] Fauron C. Casper M. Gao Y. Moore B. (1995). The maize mitochondrial genome: dynamic, yet functional. Trends Genet. 11, 228–235. doi: 10.1016/S0168-9525(00)89056-3 7638905

[B13] Graham H. N. (1992). Green tea composition, consumption, and polyphenol chemistry. Prev. Med. 21, 334–350. doi: 10.1016/0091-7435(92)90041-f 1614995

[B14] Guoben C. (1987). A discussion on the genetic relationship and taxonomic position of tea resources in Hunan. J. Hunan Agri. Univ. 1987 (03), 71–79. doi: 10.13331/j.cnki.jhau.1987.03.013

[B15] Gualberto J. M. Mileshina D. Wallet C. Niazi A. K. Weber-Lotfi F. Dietrich A. (2014). The plant mitochondrial genome: dynamics and maintenance. Biochimie 100, 107–120. doi: 10.1016/j.biochi.2013.09.016 24075874

[B16] Guo W. Zhu A. Fan W. Mower J. P. (2017). Complete mitochondrial genomes from the ferns ophioglossum californicum and psilotum nudum are highly repetitive with the largest organellar introns. New Phytol. 213, 391–403. doi: 10.1111/nph.14135 27539928

[B17] Huan G. Jie K. (2005). Distribution characteristics and biological function of tandem repeat sequences in the genomes of different organisms. Zool. Res. 26, 555–564. doi: 10.3321/j.issn:0254-5853.2005.05.017

[B18] Koren S. Walenz B. P. Berlin K. Miller J. R. Bergman N. H. Phillippy A. M. (2017). Canu: scalable and accurate long-read assembly *via* adaptive k-mer weighting and repeat separation. Genome Res. 27, 722–736. doi: 10.1101/gr.215087.116 28298431 PMC5411767

[B19] Kozik A. Rowan B. A. Lavelle D. Berke L. Schranz M. E. Michelmore R. W. . (2019). The alternative reality of plant mitochondrial DNA: One ring does not rule them all. PloS Genet. 15, e1008373. doi: 10.1371/journal.pgen.1008373 31469821 PMC6742443

[B20] Kurtz S. Choudhuri J. V. Ohlebusch E. Schleiermacher C. Stoye J. Giegerich R. (2001). REPuter: the manifold applications of repeat analysis on a genomic scale. Nucleic Acids Res. 29, 4633–4642. doi: 10.1093/nar/29.22.4633 11713313 PMC92531

[B21] Li J. Lv Q. Zhang X.-M. Han H.-L. Zhang A.-B. (2021). Characterization and phylogenetic analysis of the complete mitochondrial genome of laelia suffusa (Lepidoptera: Erebidae, lymantriinae). J. Insect Sci. Online 21, 5. doi: 10.1093/jisesa/ieaa138 PMC7799433 33428744

[B22] Malek O. Lättig K. Hiesel R. Brennicke A. Knoop V. (1996). RNA Editing in bryophytes and a molecular phylogeny of land plants. EMBO J. 15, 1403–1411. doi: 10.1002/j.1460-2075.1996.tb00482.x 8635473 PMC450045

[B23] Maréchal A. Brisson N. (2010). Recombination and the maintenance of plant organelle genome stability. New Phytol. 186, 299–317. doi: 10.1111/j.1469-8137.2010.03195.x 20180912

[B24] McCarty M. F. Assanga S. I. Lujan L. L. (2020). Flavones and flavonols may have clinical potential as CK2 inhibitors in cancer therapy. Med. Hypotheses 141, 109723. doi: 10.1016/j.mehy.2020.109723 32305811

[B25] Mower J. P. Sloan D. B. Alverson A. J. (2012). “Plant mitochondrial genome diversity: The genomics revolution,” in Plant genome diversity volume 1: Plant genomes, their residents, and their evolutionary dynamics. Eds. Wendel J. F. Greilhuber J. Dolezel J. Leitch I. J. (Vienna: Springer), 123–144. doi: 10.1007/978-3-7091-1130-7_9

[B26] Nguyen V. B. Linh Giang V. N. Waminal N. E. Park H.-S. Kim N.-H. Jang W. . (2020). Comprehensive comparative analysis of chloroplast genomes from seven panax species and development of an authentication system based on species-unique single nucleotide polymorphism markers. J. Ginseng Res. 44, 135–144. doi: 10.1016/j.jgr.2018.06.003 32148396 PMC7033337

[B27] Park S. Ruhlman T. A. Sabir J. S. M. Mutwakil M. H. Z. Baeshen M. N. Sabir M. J. . (2014). Complete sequences of organelle genomes from the medicinal plant rhazya stricta (Apocynaceae) and contrasting patterns of mitochondrial genome evolution across asterids. BMC Genomics 15, 405. doi: 10.1186/1471-2164-15-405 24884625 PMC4045975

[B28] Powell W. Machray G. C. Provan J. (1996). Polymorphism revealed by simple sequence repeats. Trends Plant Sci. 1, 215–222. doi: 10.1016/1360-1385(96)86898-1

[B29] Qiao Y. Zhang X. Li Z. Song Y. Sun Z. (2022). Assembly and comparative analysis of the complete mitochondrial genome of bupleurum chinense DC. BMC Genomics 23, 664. doi: 10.1186/s12864-022-08892-z 36131243 PMC9490909

[B30] Qiu L. Yang C. Tian B. Yang J.-B. Liu A. (2010). Exploiting EST databases for the development and characterization of EST-SSR markers in castor bean (Ricinus communis l.). BMC Plant Biol. 10, 278. doi: 10.1186/1471-2229-10-278 21162723 PMC3017068

[B31] Sazanov L. A. Burrows P. A. Nixon P. J. (1998). The plastid ndh genes code for an NADH-specific dehydrogenase: Isolation of a complex I analogue from pea thylakoid membranes. Proc. Natl. Acad. Sci. 95, 1319–1324. doi: 10.1073/pnas.95.3.1319 9448329 PMC18756

[B32] Shearman J. R. Sonthirod C. Naktang C. Pootakham W. Yoocha T. Sangsrakru D. . (2016). The two chromosomes of the mitochondrial genome of a sugarcane cultivar: assembly and recombination analysis using long PacBio reads. Sci. Rep. 6, 31533. doi: 10.1038/srep31533 27530092 PMC4987617

[B33] Sloan D. B. Alverson A. J. Chuckalovcak J. P. Wu M. McCauley D. E. Palmer J. D. . (2012). Rapid evolution of enormous, multichromosomal genomes in flowering plant mitochondria with exceptionally high mutation rates. PloS Biol. 10, e1001241. doi: 10.1371/journal.pbio.1001241 22272183 PMC3260318

[B34] Stamatakis A. (2014). RAxML version 8: a tool for phylogenetic analysis and post-analysis of large phylogenies. Bioinforma. Oxf. Engl. 30, 1312–1313. doi: 10.1093/bioinformatics/btu033 PMC3998144 24451623

[B35] Sun Z. Wan D.-G. Murphy R. W. Ma L. Zhang X.-S. Huang D.-W. (2009). Comparison of base composition and codon usage in insect mitochondrial genomes. Genes Genomics 31, 65–71. doi: 10.1007/BF03191139

[B36] Unseld M. Marienfeld J. R. Brandt P. Brennicke A. (1997). The mitochondrial genome of arabidopsis thaliana contains 57 genes in 366,924 nucleotides. Nat. Genet. 15, 57–61. doi: 10.1038/ng0197-57 8988169

[B37] Wang D. Zhang Y. Zhang Z. Zhu J. Yu J. (2010). KaKs_Calculator 2.0: a toolkit incorporating gamma-series methods and sliding window strategies. Genomics Proteomics Bioinf. 8, 77–80. doi: 10.1016/S1672-0229(10)60008-3 PMC5054116 20451164

[B38] Wei S. Wang X. Bi C. Xu Y. Wu D. Ye N. (2016). Assembly and analysis of the complete salix purpurea l. (Salicaceae) mitochondrial genome sequence. SpringerPlus 5, 1894. doi: 10.1186/s40064-016-3521-6 27843751 PMC5084139

[B39] Wright F. (1990). The “effective number of codons” used in a gene. Gene 87, 23–29. doi: 10.1016/0378-1119(90)90491-9 2110097

[B40] Wu Z.-Q. Ge S. (2012). The phylogeny of the BEP clade in grasses revisited: Evidence from the whole-genome sequences of chloroplasts. Mol. Phylogenet. Evol. 62, 573–578. doi: 10.1016/j.ympev.2011.10.019 22093967

[B41] Xiao L. Xu X. Zhang F. Wang M. Xu Y. Tang D. . (2017). The mitochondria-targeted antioxidant MitoQ ameliorated tubular injury mediated by mitophagy in diabetic kidney disease *via* Nrf2/PINK1. Redox Biol. 11, 297–311. doi: 10.1016/j.redox.2016.12.022 28033563 PMC5196243

[B42] Xinghui L. Gesheng X. Xingyan C. Benqing S. Dehua L. Junwu L. (1998). Evaluation of tea plant resources in Hunan [J]. Guangxi Plants 1998 (02), 14–19.

[B43] Xu J. Luo H. Nie S. Zhang R.-G. Mao J.-F. (2021). The complete mitochondrial and plastid genomes of rhododendron simsii, an important parent of widely cultivated azaleas. Mitochondrial DNA Part B Resour. 6, 1197–1199. doi: 10.1080/23802359.2021.1903352 PMC7995824 33796782

[B44] Zhang L. Ho C.-T. Zhou J. Santos J. S. Armstrong L. Granato D. (2019). Chemistry and biological activities of processed camellia sinensis teas: A comprehensive review. Compr. Rev. Food Sci. Food Saf. 18, 1474–1495. doi: 10.1111/1541-4337.12479 33336903

[B45] Zhang W. Li L. Li G. (2018). Characterization of the complete chloroplast genome of shrubby sophora (Sophora flavescens ait.). Mitochondrial DNA Part B Resour. 3, 1282–1283. doi: 10.1080/23802359.2018.1532839 PMC7800985 33490578

[B46] Zubaer A. Wai A. Hausner G. (2018). The mitochondrial genome of endoconidiophora resinifera is intron rich. Sci. Rep. 8, 17591. doi: 10.1038/s41598-018-35926-y 30514960 PMC6279837

