# Supplementary material for: Complete mitochondrial genome assembly and comparison of Camellia sinensis var. Assamica cv. Duntsa
Source: Front Plant Sci. 2023 Jan 19;14:1117002. doi: 10.3389/fpls.2023.1117002 (PMC9893290; doi:10.3389/fpls.2023.1117002)
Supplement: Supplementary file 1 [file DataSheet_1.docx]

Supplementary Material

Complete mitochondrial genome assembly and

comparison of *Camellia sinensis var. Duntsa*

Jin Li^1,2^,Han Tang^2^,Hua Luo^2^,Jun Tang^2^,Ni Zhong^1,3^,Lizheng Xiao^1^*

^1^Key Laboratory of Tea Science of Ministry of Education, National Research Center of Engineering and Technology for Utilization of Botanical Functional Ingredients, Co-Innovation Center of Education Ministry, Changsha 410125, China,^2^Institute of Tea Research, Shaoyang Academy of Agricultural Sciences., Shaoyang 422000, China ,^3^Institute of Tea Research, Hunan Academy of Agricultural Sciences, Changsha 410125, China.

* Correspondence:

Corresponding Author,Lizheng Xiao

email@uni.edu,1369949056@qq.com

# Supplementary Figures and Tables

## Supplementary Figures

**A:
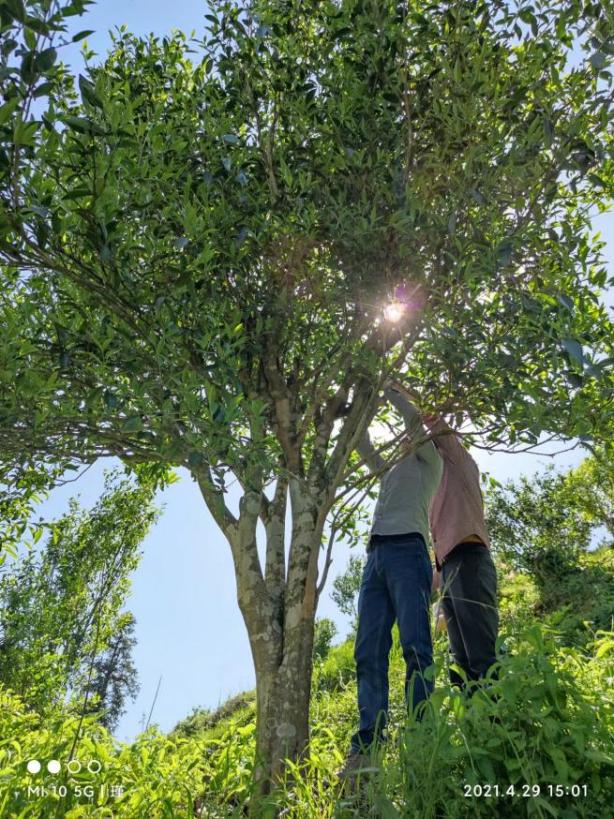
 B:
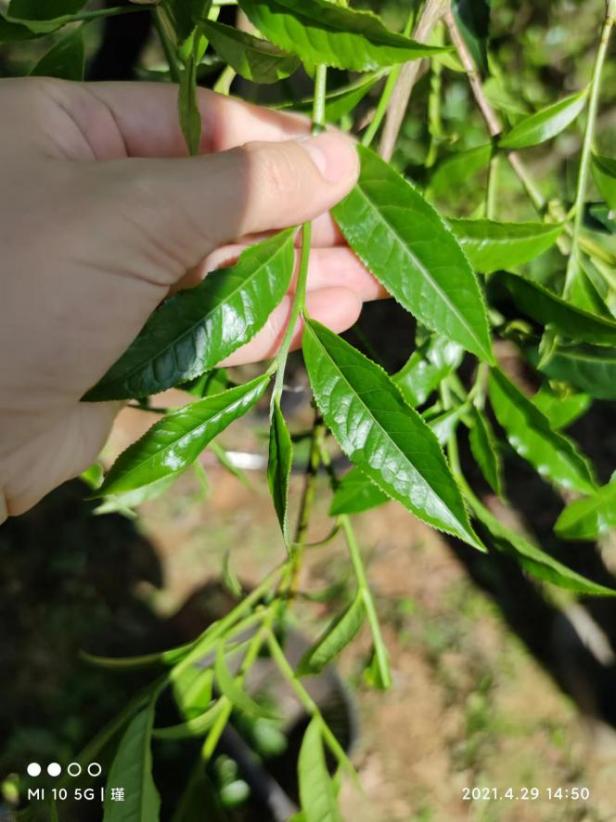
**

**Supplementary Figure 1.** Phenotypic characteristics of *C.duntsa.* **A**:The tree shape of *C.duntsa*; **B**:The leaves of *C.duntsa*.


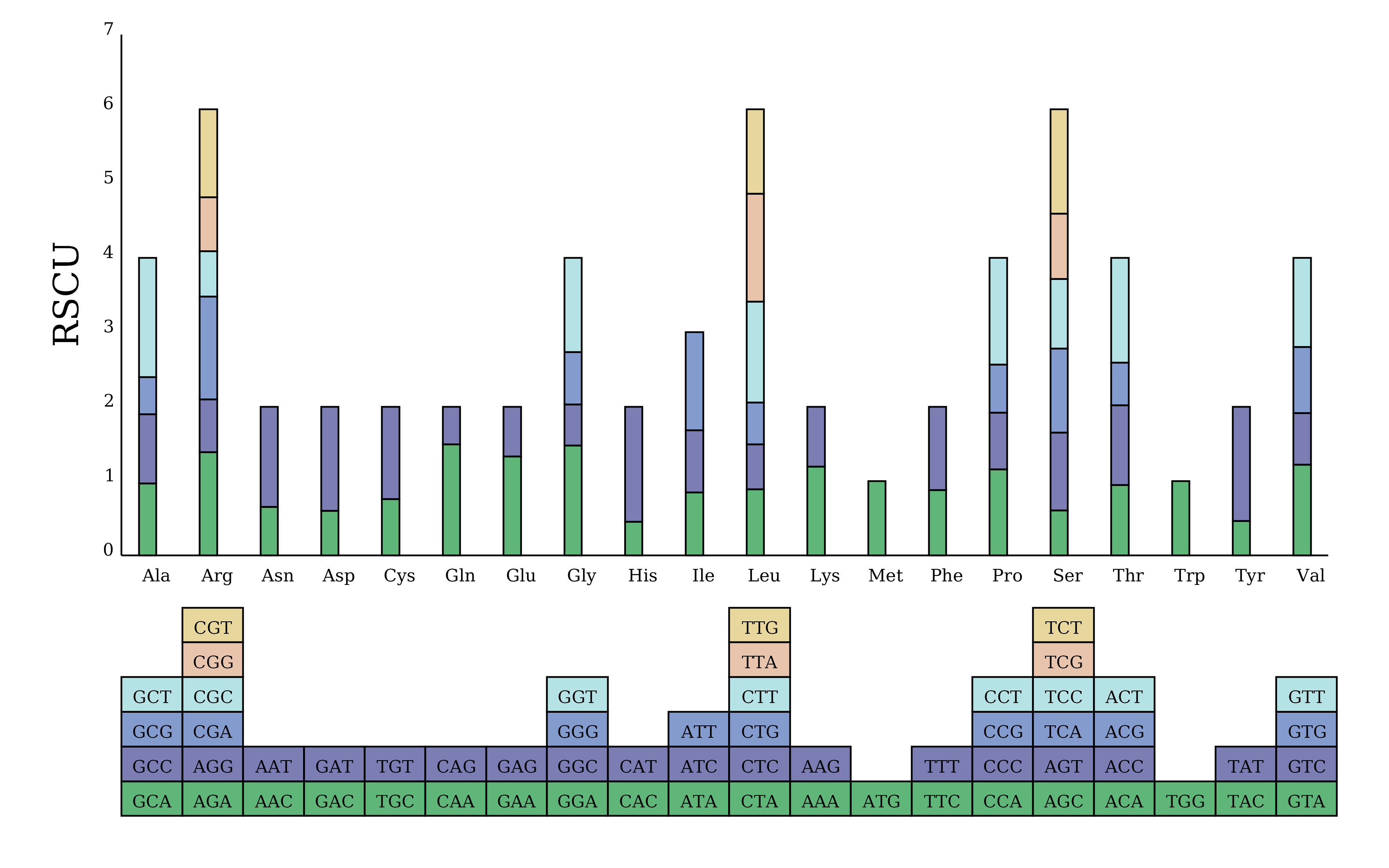


**Supplementary Figure 2.** *C.duntsa* RSCU. X-axis shows codon families. RSCU values are the number of times a codon is seen relative to the number expected for a uniform synonymous codon usage.


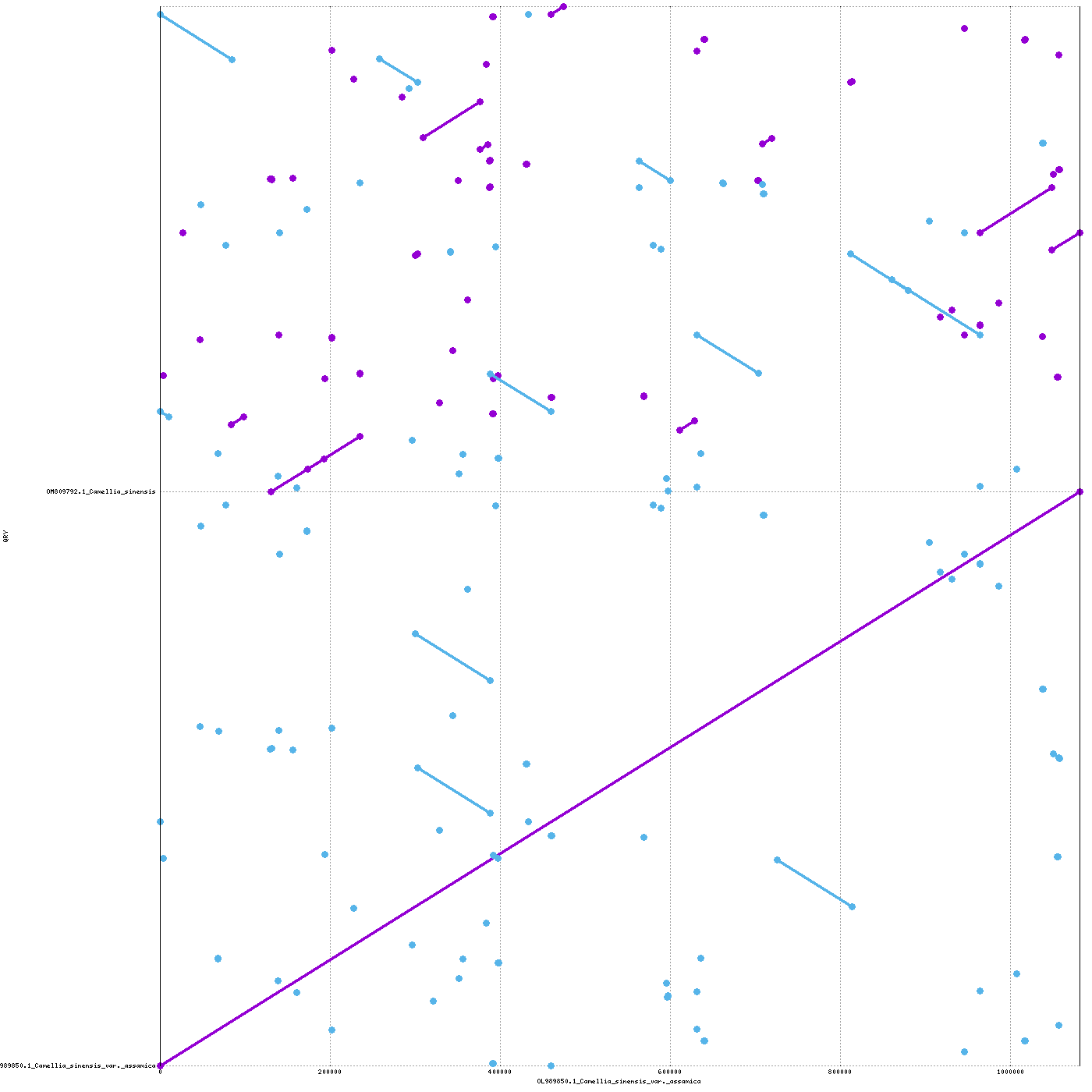


**Supplementary Figure 3.** *C.duntsa* and *C.sinensis*(OM809792) mitochondrial genome collinearity (OM809792). Purple bars represent reversed homologous high-scoring segment pairs.


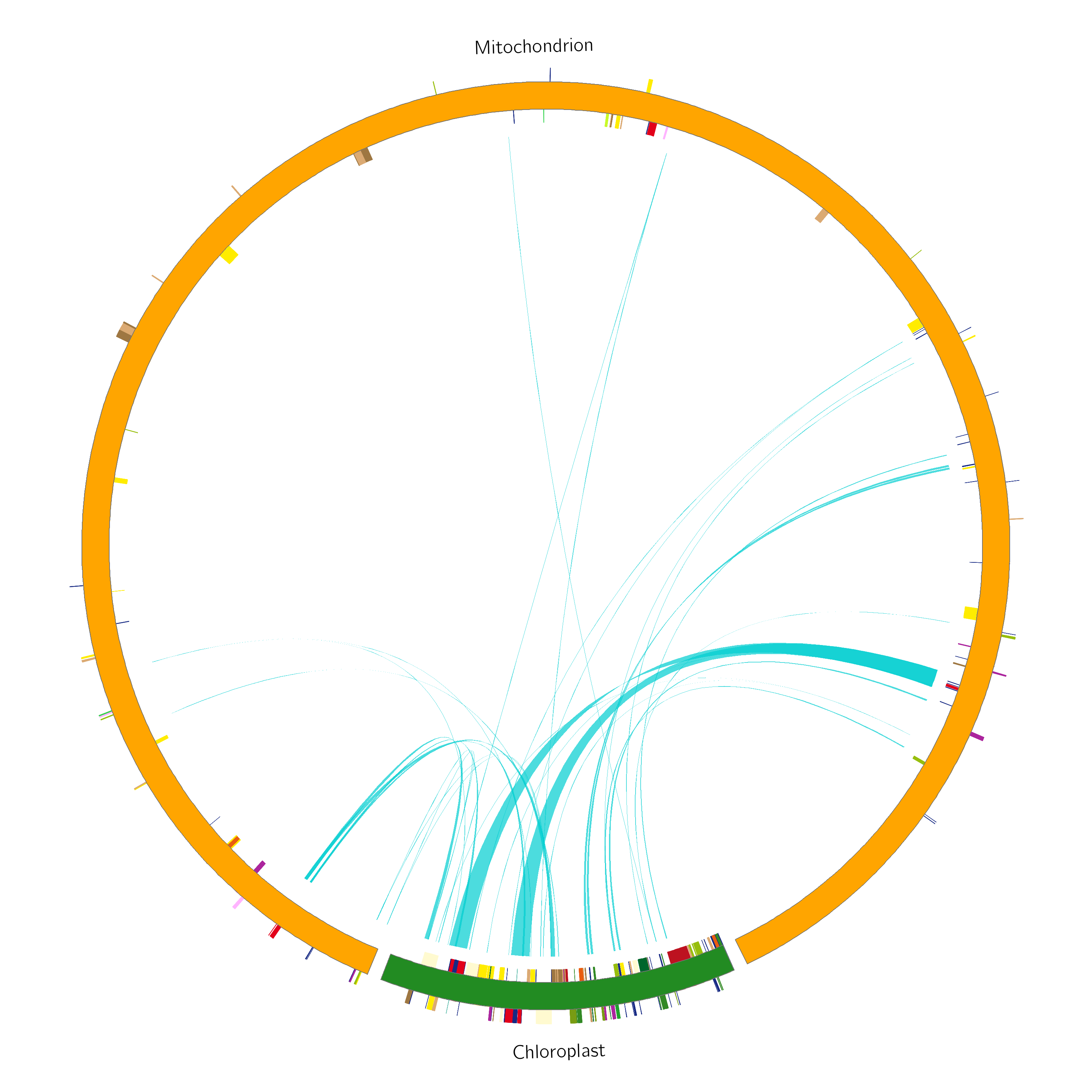


**Supplementary Figure 4.** Chloroplast and mitochondrial genomes share a homologous region. Green indicates the *C.duntsa* chloroplast genome, and yellow represents the mitochondrial genome. The blue circle segment connects the gene fragments' start and end sites. The blue line's width represents the transferred fragment.

## Supplementary Tables

| **Sample name** | **Next-generation sequencing platform** | | | | | **Third-generation sequencing platform** | | | |
| --- | --- | --- | --- | --- | --- | --- | --- | --- | --- |
|  | **Clean reads (bp)** | **Clean bases** | **Read length (bp)** | **Q30 (%)** | **GC (%)** | **Total pass reads** | **Total pass bases (GB)** | **Meanlength (bp)** | **N50 length (bp)** |
| *Camellia sinensis var. Assamica cv.Duntsa* | 26,855,210 | 8,056,563,000 | 150 | 90.62 | 41.94% | 1,034,825 | 50.883 | 12,665 | 24,205 |

**Supplementary Table 1.** Summary of Sequencing statistics

**Supplementary Table 2.** The stop codes of protein-coding genes in *C.duntsa*  mitochondrial genome

| Stop codon | Number | Percentage |
| --- | --- | --- |
| TAG | 9 | 19.15% |
| TGA | 16 | 34.04% |
| TAA | 22 | 46.81% |
| Total | 47 | 100.00% |

**Supplementary Table 3.** Relative synonymous codon usage values of the four tea plant species species mitochondrial genomes

|  |  | **OL989850** | | **NC043914** | | **OM809792** | | **MK574877** | |
| --- | --- | --- | --- | --- | --- | --- | --- | --- | --- |
| Codon | AA | No. | RSCU | No. | RSCU | No. | RSCU | No. | RSCU |
| UAA | Ter | 18 | 1.5 | 15 | 1.5517 | 19 | 1.6286 | 24 | 1.3091 |
| UAG | Ter | 7 | 0.5833 | 5 | 0.5172 | 6 | 0.5143 | 17 | 0.9273 |
| UGA | Ter | 11 | 0.9167 | 9 | 0.931 | 10 | 0.8571 | 14 | 0.7636 |
| GCA | Ala | 171 | 0.9785 | 122 | 0.9088 | 154 | 0.9716 | 124 | 0.9612 |
| GCC | Ala | 162 | 0.927 | 131 | 0.9758 | 150 | 0.9464 | 119 | 0.9225 |
| GCG | Ala | 87 | 0.4979 | 73 | 0.5438 | 77 | 0.4858 | 75 | 0.5814 |
| GCU | Ala | 279 | 1.5966 | 211 | 1.5717 | 253 | 1.5962 | 198 | 1.5349 |
| UGC | Cys | 58 | 0.7484 | 46 | 0.7797 | 51 | 0.7183 | 52 | 0.7879 |
| UGU | Cys | 97 | 1.2516 | 72 | 1.2203 | 91 | 1.2817 | 80 | 1.2121 |
| GAC | Asp | 104 | 0.6082 | 74 | 0.6116 | 90 | 0.5788 | 85 | 0.6391 |
| GAU | Asp | 238 | 1.3918 | 168 | 1.3884 | 221 | 1.4212 | 181 | 1.3609 |
| GAA | Glu | 303 | 1.3319 | 211 | 1.3146 | 273 | 1.3582 | 265 | 1.3625 |
| GAG | Glu | 152 | 0.6681 | 110 | 0.6854 | 129 | 0.6418 | 124 | 0.6375 |
| UUC | Phe | 298 | 0.8882 | 222 | 0.8672 | 270 | 0.8668 | 237 | 0.9222 |
| UUU | Phe | 373 | 1.1118 | 290 | 1.1328 | 353 | 1.1332 | 277 | 1.0778 |
| GGA | Gly | 282 | 1.4611 | 195 | 1.4745 | 260 | 1.5182 | 213 | 1.4083 |
| GGC | Gly | 105 | 0.544 | 68 | 0.5142 | 89 | 0.5197 | 92 | 0.6083 |
| GGG | Gly | 141 | 0.7306 | 94 | 0.7108 | 114 | 0.6657 | 112 | 0.7405 |
| GGU | Gly | 244 | 1.2642 | 172 | 1.3006 | 222 | 1.2964 | 188 | 1.243 |
| CAC | His | 60 | 0.4461 | 46 | 0.4946 | 58 | 0.4622 | 52 | 0.4976 |
| CAU | His | 209 | 1.5539 | 140 | 1.5054 | 193 | 1.5378 | 157 | 1.5024 |
| AUA | Ile | 233 | 0.8462 | 165 | 0.787 | 203 | 0.7971 | 180 | 0.806 |
| AUC | Ile | 231 | 0.839 | 170 | 0.8108 | 215 | 0.8442 | 203 | 0.909 |
| AUU | Ile | 362 | 1.3148 | 294 | 1.4022 | 346 | 1.3586 | 287 | 1.2851 |
| AAA | Lys | 282 | 1.1899 | 181 | 1.1565 | 243 | 1.215 | 236 | 1.154 |
| AAG | Lys | 192 | 0.8101 | 132 | 0.8435 | 157 | 0.785 | 173 | 0.846 |
| CUA | Leu | 162 | 0.882 | 132 | 0.9135 | 154 | 0.8902 | 113 | 0.7713 |
| CUC | Leu | 114 | 0.6207 | 80 | 0.5536 | 103 | 0.5954 | 101 | 0.6894 |
| CUG | Leu | 104 | 0.5662 | 78 | 0.5398 | 96 | 0.5549 | 86 | 0.587 |
| CUU | Leu | 245 | 1.3339 | 197 | 1.3633 | 232 | 1.341 | 203 | 1.3857 |
| UUA | Leu | 261 | 1.4211 | 206 | 1.4256 | 250 | 1.4451 | 206 | 1.4061 |
| UUG | Leu | 216 | 1.176 | 174 | 1.2042 | 203 | 1.1734 | 170 | 1.1604 |
| AUG | Met | 286 | 1 | 206 | 1 | 271 | 1 | 217 | 1 |
| AAC | Asn | 113 | 0.657 | 77 | 0.6235 | 99 | 0.6429 | 109 | 0.7315 |
| AAU | Asn | 231 | 1.343 | 170 | 1.3765 | 209 | 1.3571 | 189 | 1.2685 |
| CCA | Pro | 180 | 1.1632 | 127 | 1.0972 | 162 | 1.1592 | 139 | 1.1488 |
| CCC | Pro | 119 | 0.769 | 94 | 0.8121 | 106 | 0.7585 | 99 | 0.8182 |
| CCG | Pro | 96 | 0.6204 | 74 | 0.6393 | 84 | 0.6011 | 76 | 0.6281 |
| CCU | Pro | 224 | 1.4475 | 168 | 1.4514 | 207 | 1.4812 | 170 | 1.405 |
| CAA | Gln | 224 | 1.4933 | 159 | 1.4521 | 203 | 1.4982 | 186 | 1.4646 |
| CAG | Gln | 76 | 0.5067 | 60 | 0.5479 | 68 | 0.5018 | 68 | 0.5354 |
| AGA | Arg | 177 | 1.3955 | 108 | 1.218 | 151 | 1.3583 | 154 | 1.4348 |
| AGG | Arg | 92 | 0.7254 | 63 | 0.7105 | 79 | 0.7106 | 86 | 0.8012 |
| CGA | Arg | 172 | 1.3561 | 127 | 1.4323 | 155 | 1.3943 | 140 | 1.3043 |
| CGC | Arg | 77 | 0.6071 | 58 | 0.6541 | 65 | 0.5847 | 69 | 0.6429 |
| CGG | Arg | 91 | 0.7175 | 69 | 0.7782 | 80 | 0.7196 | 78 | 0.7267 |
| CGU | Arg | 152 | 1.1984 | 107 | 1.2068 | 137 | 1.2324 | 117 | 1.0901 |
| AGC | Ser | 101 | 0.6024 | 74 | 0.5952 | 88 | 0.5733 | 87 | 0.6335 |
| AGU | Ser | 173 | 1.0318 | 128 | 1.0295 | 162 | 1.0554 | 139 | 1.0121 |
| UCA | Ser | 190 | 1.1332 | 145 | 1.1662 | 181 | 1.1792 | 158 | 1.1505 |
| UCC | Ser | 161 | 0.9602 | 127 | 1.0214 | 148 | 0.9642 | 122 | 0.8883 |
| UCG | Ser | 150 | 0.8946 | 112 | 0.9008 | 136 | 0.886 | 123 | 0.8956 |
| UCU | Ser | 231 | 1.3777 | 160 | 1.2869 | 206 | 1.342 | 195 | 1.4199 |
| ACA | Thr | 130 | 0.9403 | 103 | 1.0274 | 123 | 0.9685 | 111 | 0.9911 |
| ACC | Thr | 145 | 1.0488 | 95 | 0.9476 | 131 | 1.0315 | 124 | 1.1071 |
| ACG | Thr | 84 | 0.6076 | 63 | 0.6284 | 71 | 0.5591 | 65 | 0.5804 |
| ACU | Thr | 194 | 1.4033 | 140 | 1.3965 | 183 | 1.4409 | 148 | 1.3214 |
| GUA | Val | 200 | 1.1976 | 145 | 1.1934 | 186 | 1.2039 | 152 | 1.1343 |
| GUC | Val | 120 | 0.7186 | 93 | 0.7654 | 110 | 0.712 | 94 | 0.7015 |
| GUG | Val | 148 | 0.8862 | 101 | 0.8313 | 130 | 0.8414 | 125 | 0.9328 |
| GUU | Val | 200 | 1.1976 | 147 | 1.2099 | 192 | 1.2427 | 165 | 1.2313 |
| UGG | Trp | 161 | 1 | 115 | 1 | 148 | 1 | 134 | 1 |
| UAC | Tyr | 76 | 0.4677 | 57 | 0.4831 | 69 | 0.4585 | 62 | 0.5 |
| UAU | Tyr | 249 | 1.5323 | 179 | 1.5169 | 232 | 1.5415 | 186 | 1.5 |

**Supplementary Table 4.** Effective number of codons values of the genes from four tea plant species mitochondrial genomes

| **Species** | **GC all content （%）** | **GC1 content （%）** | **GC2 content （%）** | **GC3 content （%）** | **ENC** | **gene** |
| --- | --- | --- | --- | --- | --- | --- |
| OL989850 | 39.88 | 41.31 | 44.17 | 34.15 | 49.32 | nad2 |
| OL989850 | 37.95 | 39.6 | 42.57 | 31.68 | 46.62 | nad4L |
| OL989850 | 43.35 | 46.63 | 43.01 | 40.41 | 61 | atp4 |
| OL989850 | 43.82 | 47.41 | 48.61 | 35.46 | 48.97 | ccmC |
| OL989850 | 43.42 | 47.9 | 45.69 | 36.67 | 52.74 | cox1 |
| OL989850 | 46.09 | 49.43 | 45.33 | 43.51 | 55.96 | ccmFC |
| OL989850 | 42.02 | 53.99 | 41.41 | 30.67 | 49.59 | nad1 |
| OL989850 | 52.39 | 52.9 | 45.88 | 58.38 | 54.61 | matR |
| OL989850 | 40.34 | 42.02 | 45.38 | 33.61 | 46.85 | nad3 |
| OL989850 | 44.71 | 53.97 | 49.21 | 30.95 | 54.2 | rps12 |
| OL989850 | 41.25 | 37.5 | 38.75 | 47.5 | 50.22 | atp8 |
| OL989850 | 43.48 | 53.01 | 43.61 | 33.83 | 52.12 | cox3 |
| OL989850 | 39.02 | 39.53 | 32.56 | 44.96 | 46.92 | sdh4 |
| OL989850 | 39.95 | 42.24 | 41.88 | 35.74 | 54.6 | rps4 |
| OL989850 | 39.97 | 43.2 | 37.38 | 39.32 | 52.96 | nad6 |
| OL989850 | 41.18 | 43.67 | 43.96 | 35.92 | 50.18 | nad5 |
| OL989850 | 45.68 | 49.38 | 53.09 | 34.57 | 35.73 | atp9 |
| OL989850 | 50.89 | 53.65 | 54.83 | 44.18 | 54.51 | rpl2 |
| OL989850 | 37.23 | 36.36 | 45.45 | 29.87 | 49.78 | rps19 |
| OL989850 | 43.56 | 45.21 | 41.49 | 43.97 | 56.65 | rps3 |
| OL989850 | 44.57 | 50.58 | 51.74 | 31.4 | 51.1 | rpl16 |
| OL989850 | 42.51 | 51.68 | 40.27 | 35.57 | 59.99 | rps7 |
| OL989850 | 43.97 | 55.44 | 44.81 | 31.65 | 46.98 | nad7 |
| OL989850 | 43.07 | 46.53 | 41.09 | 41.58 | 48.29 | rps1 |
| OL989850 | 44.57 | 50.58 | 51.74 | 31.4 | 51.1 | rpl16 |
| OL989850 | 43.56 | 45.21 | 41.49 | 43.97 | 56.65 | rps3 |
| OL989850 | 37.23 | 36.36 | 45.45 | 29.87 | 49.78 | rps19 |
| OL989850 | 50.89 | 53.65 | 54.83 | 44.18 | 54.51 | rpl2 |
| OL989850 | 45.68 | 49.38 | 53.09 | 34.57 | 35.73 | atp9 |
| OL989850 | 37.69 | 38.32 | 35.51 | 39.25 | 46.53 | sdh3 |
| OL989850 | 41.62 | 50.76 | 41.62 | 32.49 | 50.53 | cob |
| OL989850 | 41.58 | 43.56 | 45.54 | 35.64 | 54.4 | rps14 |
| OL989850 | 42.73 | 48.94 | 37.77 | 41.49 | 54.99 | rpl5 |
| OL989850 | 37.61 | 47.01 | 41.03 | 24.79 | 37.79 | rps13 |
| OL989850 | 40.78 | 52.27 | 39.77 | 30.3 | 48.05 | cox2 |
| OL989850 | 43.56 | 45.21 | 41.49 | 43.97 | 56.65 | rps3 |
| OL989850 | 37.23 | 36.36 | 45.45 | 29.87 | 49.78 | rps19 |
| OL989850 | 45.68 | 49.38 | 53.09 | 34.57 | 35.73 | atp9 |
| OL989850 | 42.23 | 51.31 | 41.36 | 34.03 | 55.85 | nad9 |
| OL989850 | 38.95 | 37.89 | 46.32 | 32.63 | 48.14 | rps19 |
| OL989850 | 39.85 | 44.96 | 38.91 | 35.69 | 52.42 | nad4 |
| OL989850 | 38.5 | 46.52 | 37.97 | 31.01 | 48.99 | atp6 |
| OL989850 | 45.11 | 46.55 | 41.38 | 47.41 | 47.78 | mttB |
| OL989850 | 42.03 | 46.38 | 45.41 | 34.3 | 54.42 | ccmB |
| OL989850 | 42.33 | 47.24 | 42.94 | 36.81 | 52.2 | rpl10 |
| OL989850 | 47.3 | 51.75 | 48.91 | 41.24 | 53.94 | ccmFN |
| OL989850 | 44.97 | 57.84 | 42.75 | 34.31 | 52.32 | atp1 |
| NC043914 | 41.15 | 43.61 | 44.06 | 35.79 | 50.38 | nad5 |
| NC043914 | 37.61 | 47.01 | 41.03 | 24.79 | 37.79 | rps13 |
| NC043914 | 46.67 | 50.67 | 54.67 | 34.67 | 33.62 | atp9 |
| NC043914 | 39.97 | 43.2 | 37.38 | 39.32 | 52.96 | nad6 |
| NC043914 | 42.38 | 54.27 | 41.77 | 31.1 | 50.5 | nad1 |
| NC043914 | 37.61 | 47.01 | 41.03 | 24.79 | 37.79 | rps13 |
| NC043914 | 40.78 | 52.27 | 39.77 | 30.3 | 48.05 | cox2 |
| NC043914 | 39.3 | 37.89 | 46.32 | 33.68 | 48.14 | rps16 |
| NC043914 | 39.41 | 39.7 | 44.21 | 34.33 | 49.37 | nad2 |
| NC043914 | 44.97 | 53.97 | 49.21 | 31.75 | 54.81 | rps12 |
| NC043914 | 40.34 | 42.02 | 45.38 | 33.61 | 46.85 | nad3 |
| NC043914 | 44.84 | 50 | 39.88 | 44.64 | 49.82 | rps1 |
| NC043914 | 52.4 | 52.61 | 46.01 | 58.59 | 54.69 | matR |
| NC043914 | 47.04 | 51.55 | 48.29 | 41.27 | 53.94 | ccmFn |
| NC043914 | 43.82 | 47.41 | 48.61 | 35.46 | 48.97 | ccmC |
| NC043914 | 43.35 | 46.63 | 43.01 | 40.41 | 61 | atp4 |
| NC043914 | 38.83 | 41.76 | 42.86 | 31.87 | 45.18 | nad4l |
| NC043914 | 44.97 | 57.84 | 42.75 | 34.31 | 52.32 | atp1 |
| NC043914 | 42.28 | 51.68 | 40.27 | 34.9 | 59.08 | rps7 |
| NC043914 | 42.54 | 47.24 | 43.56 | 36.81 | 52.34 | rpl10 |
| NC043914 | 42.03 | 46.38 | 45.41 | 34.3 | 54.42 | ccmB |
| NC043914 | 42.75 | 42.75 | 42.03 | 43.48 | 51.54 | mttB |
| NC043914 | 38.5 | 46.52 | 37.97 | 31.01 | 49.09 | atp6 |
| NC043914 | 39.87 | 45.05 | 38.99 | 35.56 | 52.42 | nad4 |
| NC043914 | 54.81 | 56.67 | 57.78 | 50.00 | 57.57 | orf115b |
| NC043914 | 45.86 | 49.44 | 44.97 | 43.18 | 55.82 | ccmFc |
| NC043914 | 37.38 | 38.32 | 34.58 | 39.25 | 45.73 | sdh3 |
| NC043914 | 53.76 | 51.61 | 54.84 | 54.84 | 43.76 | orf100 |
| NC043914 | 42.73 | 48.94 | 37.77 | 41.49 | 54.99 | rpl5 |
| NC043914 | 41.58 | 43.56 | 45.54 | 35.64 | 54.4 | rps14 |
| NC043914 | 41.46 | 50.76 | 41.37 | 32.23 | 50.42 | cob |
| NC043914 | 39.3 | 37.89 | 46.32 | 33.68 | 48.14 | rps16 |
| OM809792 | 40.34 | 42.02 | 45.38 | 33.61 | 46.85 | nad3 |
| OM809792 | 44.71 | 53.97 | 49.21 | 30.95 | 54.2 | rps12 |
| OM809792 | 39.88 | 41.31 | 44.17 | 34.15 | 49.32 | nad2 |
| OM809792 | 41.25 | 37.5 | 38.75 | 47.5 | 50.22 | atp8 |
| OM809792 | 43.48 | 53.01 | 43.61 | 33.83 | 52.12 | cox3 |
| OM809792 | 39.02 | 39.53 | 32.56 | 44.96 | 46.92 | sdh4 |
| OM809792 | 39.83 | 42.24 | 41.52 | 35.74 | 54.54 | rps4 |
| OM809792 | 39.97 | 43.2 | 37.38 | 39.32 | 52.96 | nad6 |
| OM809792 | 41.18 | 43.67 | 43.96 | 35.92 | 50.18 | nad5 |
| OM809792 | 42.02 | 53.99 | 41.41 | 30.67 | 49.59 | nad1 |
| OM809792 | 52.39 | 52.9 | 45.88 | 58.38 | 54.61 | matR |
| OM809792 | 43.82 | 47.41 | 48.61 | 35.46 | 48.97 | ccmC |
| OM809792 | 43.35 | 46.63 | 43.01 | 40.41 | 61 | atp4 |
| OM809792 | 37.95 | 39.6 | 42.57 | 31.68 | 46.62 | nad4L |
| OM809792 | 37.63 | 47.35 | 26.14 | 39.39 | 56.73 | atp8 |
| OM809792 | 43.23 | 46.53 | 41.09 | 42.08 | 48.67 | rps1 |
| OM809792 | 43.97 | 55.44 | 44.81 | 31.65 | 46.98 | nad7 |
| OM809792 | 42.51 | 51.68 | 40.27 | 35.57 | 59.99 | rps7 |
| OM809792 | 40.78 | 52.27 | 39.77 | 30.3 | 48.05 | cox2 |
| OM809792 | 37.61 | 47.01 | 41.03 | 24.79 | 37.79 | rps13 |
| OM809792 | 42.73 | 48.94 | 37.77 | 41.49 | 54.99 | rpl5 |
| OM809792 | 41.58 | 43.56 | 45.54 | 35.64 | 54.4 | rps14 |
| OM809792 | 41.62 | 50.76 | 41.62 | 32.49 | 50.53 | cob |
| OM809792 | 38.95 | 37.89 | 46.32 | 32.63 | 48.14 | rps19 |
| OM809792 | 42.23 | 51.31 | 41.36 | 34.03 | 55.85 | nad9 |
| OM809792 | 44.97 | 57.84 | 42.75 | 34.31 | 52.32 | atp1 |
| OM809792 | 39.85 | 44.96 | 38.91 | 35.69 | 52.42 | nad4 |
| OM809792 | 38.5 | 46.52 | 37.97 | 31.01 | 48.99 | atp6 |
| OM809792 | 45.07 | 45.6 | 43.2 | 46.4 | 49.62 | mttB |
| OM809792 | 42.03 | 46.38 | 45.41 | 34.3 | 54.42 | ccmB |
| OM809792 | 42.33 | 47.24 | 42.94 | 36.81 | 51.73 | rpl10 |
| OM809792 | 47.3 | 51.75 | 48.91 | 41.24 | 53.94 | ccmFN |
| OM809792 | 46.88 | 56 | 44.21 | 40.42 | 54.63 | cox2 |
| OM809792 | 38.01 | 38.32 | 35.51 | 40.19 | 45.38 | sdh3 |
| OM809792 | 45.68 | 49.38 | 53.09 | 34.57 | 35.73 | atp9 |
| OM809792 | 37.23 | 36.36 | 45.45 | 29.87 | 49.78 | rps19 |
| OM809792 | 44.57 | 50.58 | 51.74 | 31.4 | 51.1 | rpl16 |
| OM809792 | 46.09 | 49.43 | 45.33 | 43.51 | 55.96 | ccmFC |
| OM809792 | 43.42 | 47.9 | 45.69 | 36.67 | 52.74 | cox1 |
| OM809792 | 43.82 | 47.41 | 48.61 | 35.46 | 48.97 | ccmC |
| OM809792 | 43.35 | 46.63 | 43.01 | 40.41 | 61 | atp4 |
| OM809792 | 37.95 | 39.6 | 42.57 | 31.68 | 46.62 | nad4L |
| MK574877 | 52.4 | 52.61 | 46.01 | 58.59 | 54.69 | matR |
| MK574877 | 47.3 | 51.75 | 48.91 | 41.24 | 53.94 | ccmFn |
| MK574877 | 39.87 | 45.05 | 38.99 | 35.56 | 52.42 | nad4 |
| MK574877 | 38.5 | 46.52 | 37.97 | 31.01 | 49.09 | atp6 |
| MK574877 | 45.07 | 45.6 | 43.2 | 46.4 | 49.62 | mttB |
| MK574877 | 42.03 | 46.38 | 45.41 | 34.3 | 54.42 | ccmB |
| MK574877 | 42.54 | 47.24 | 43.56 | 36.81 | 52.34 | rpl10 |
| MK574877 | 42.06 | 51.68 | 39.6 | 34.9 | 58.9 | rps7 |
| MK574877 | 44.84 | 57.65 | 42.75 | 34.12 | 52.13 | atp1 |
| MK574877 | 38.01 | 34.58 | 32.71 | 46.73 | 40.83 | sdh3 |
| MK574877 | 43.37 | 48.92 | 49.46 | 31.72 | 50.5 | rpl16 |
| MK574877 | 44.09 | 45.39 | 41.67 | 45.21 | 56.74 | rps3 |
| MK574877 | 39.09 | 38.18 | 46.36 | 32.73 | 42.78 | rps19 |
| MK574877 | 51.07 | 49.85 | 53.52 | 49.85 | 54.42 | rpl2 |
| MK574877 | 41.04 | 36.88 | 38.75 | 47.5 | 50.42 | atp8 |
| MK574877 | 43.36 | 52.63 | 43.61 | 33.83 | 52.26 | cox3 |
| MK574877 | 41.44 | 40.28 | 36.81 | 47.22 | 51.63 | sdh4 |
| MK574877 | 43.43 | 47.48 | 45.42 | 37.38 | 53.47 | cox1 |
| MK574877 | 45.66 | 54.34 | 46.78 | 35.85 | 50.6 | nad7 |
| MK574877 | 42.23 | 51.31 | 41.36 | 34.03 | 55.85 | nad9 |
| MK574877 | 38.83 | 41.76 | 42.86 | 31.87 | 45.18 | nad4L |
| MK574877 | 43.35 | 46.63 | 43.01 | 40.41 | 61 | atp4 |
| MK574877 | 43.82 | 47.41 | 48.61 | 35.46 | 48.97 | ccmC |
| MK574877 | 40.78 | 52.27 | 39.77 | 30.3 | 48.05 | cox2 |
| MK574877 | 37.61 | 47.01 | 41.03 | 24.79 | 37.79 | rps13 |
| MK574877 | 39.35 | 41.26 | 41.55 | 35.24 | 54.01 | rps4 |
| MK574877 | 39.97 | 43.2 | 37.38 | 39.32 | 52.96 | nad6 |
| MK574877 | 43 | 50.72 | 40.58 | 37.68 | 53.23 | atp9 |
| MK574877 | 40.04 | 38.09 | 42.38 | 39.65 | 54.62 | nad2 |
| MK574877 | 44.97 | 53.97 | 49.21 | 31.75 | 54.81 | rps12 |
| MK574877 | 40.34 | 42.02 | 45.38 | 33.61 | 46.85 | nad3 |
| MK574877 | 43.07 | 46.53 | 41.09 | 41.58 | 48.29 | rps1 |

**Supplementary Table 5.** Distribution of penta and hexa SSRs in *C.duntsa* mitochondrial genome

| No. | Type | SSR | size | Start | End | Location |
| --- | --- | --- | --- | --- | --- | --- |
| 1 | pentamer | (ACTAG)3 | 15 | 90324 | 90338 | nad1-intron2,matR(partical:93.33%) |
| 2 | pentamer | (AAGGC)3 | 15 | 332157 | 332171 | IGS(atp9,rpl2) |
| 3 | pentamer | (CTTTA)3 | 15 | 449600 | 449614 | IGS(rps1,rpl16-2) |
| 4 | pentamer | (TTCTA)3 | 15 | 470269 | 470283 | IGS(rps1,rpl16-2) |
| 5 | pentamer | (GCCTT)3 | 15 | 532832 | 532846 | IGS(rpl2-2,atp9-2) |
| 6 | pentamer | (AGGCA)3 | 15 | 697759 | 697773 | IGS(cox2,rps3-3) |
| 7 | pentamer | (CCTAT)3 | 15 | 698839 | 698853 | IGS(cox2,rps3-3) |
| 8 | pentamer | (ATTAG)4 | 20 | 699064 | 699083 | IGS(cox2,rps3-3) |
| 9 | pentamer | (TTCTA)3 | 15 | 719974 | 719988 | IGS(cox2,rps3-3) |
| 10 | pentamer | (GCCTT)3 | 15 | 782537 | 782551 | IGS(rps19-3,atp9-3) |
| 11 | pentamer | (TAGAG)3 | 15 | 861835 | 861849 | IGS(nad1,trnC-GCA-2) |
| 12 | pentamer | (TATAT)3 | 15 | 880386 | 880400 | IGS(trnC-GCA-2,trnQ-TTG) |
| 13 | pentamer | (TACTT)3 | 15 | 884809 | 884823 | IGS(trnC-GCA-2,trnQ-TTG) |
| 14 | pentamer | (TAGAG)3 | 15 | 917482 | 917496 | IGS(trnE-TTC,rps19-4) |
| 15 | pentamer | (TTCAT)3 | 15 | 968768 | 968782 | IGS(trnM-CAT-4,nad4) |
| 16 | pentamer | (AGATT)3 | 15 | 1029215 | 1029229 | IGS(trnI-GAT-2,ccmFN) |
| 17 | hexamer | (TTCTAA)3 | 18 | 329386 | 329403 | IGS(atp9,rpl2) |
| 18 | hexamer | (TTAGAA)3 | 18 | 535600 | 535617 | IGS(rpl2-2,atp9-2) |
| 19 | hexamer | (AATAGA)3 | 18 | 693499 | 693516 | IGS(cox2,rps3-3) |
| 20 | hexamer | (TTAGAA)3 | 18 | 785305 | 785322 | IGS(rps19-3,atp9-3) |

**Supplementary Table 6.** Distribution of perfect tandem repeats in *C.duntsa* mitochondrial genome

| NO. | Size | Repeat sequence | Copy | Percent Matches | Start | End |
| --- | --- | --- | --- | --- | --- | --- |
| 1 | 19 | TCTGAAAGATAGACTCTTT | 2 | 100 | 2865 | 2902 |
| 2 | 15 | TAAGAAGAGTAACAG | 2.5 | 100 | 13197 | 13233 |
| 3 | 12 | TCTTCGAACCTA | 2.1 | 100 | 294974 | 294998 |
| 4 | 21 | TTCTTCGTCCCTTTCTTCTGC | 2.3 | 96 | 306360 | 306408 |
| 5 | 24 | TTTTCTTTTAGAATTGTCTCCATG | 2 | 95 | 308508 | 308554 |
| 6 | 24 | ATGGAGACAACTCTAAAAGAAAAC | 2 | 95 | 556449 | 556495 |
| 7 | 21 | CGAAGAAGCAGAAGAAAGGGA | 2.3 | 96 | 558595 | 558643 |
| 8 | 24 | CCGGCGCAGGCTCAGCAGGAGGGG | 3 | 97 | 598416 | 598487 |
| 9 | 24 | ATGGAGACAACTCTAAAAGAAAAC | 2 | 95 | 806154 | 806200 |
| 10 | 21 | CGAAGAAGCAGAAGAAAGGGA | 2.3 | 96 | 808300 | 808348 |
| 11 | 39 | AATATCATGATCGGGTCGACCAGGCCAGATCATGAGTGA | 2 | 97 | 844366 | 844444 |
| 12 | 19 | AATCATTCTTCTAGGAATG | 3.2 | 97 | 1037355 | 1037414 |
| 13 | 22 | CGAAGCCTAGAACCAGTGATGA | 2 | 95 | 1045294 | 1045337 |

**Supplementary Table 7.** Prediction of RNA editing sites

| Type | RNA -editing | Number | Percentage |
| --- | --- | --- | --- |
| hydrophobic | CTT (L) => TTT (F) | 13 | 30.84% |
|  | CCG (P) => CTG (L) | 36 |  |
|  | CCA (P) => CTA (L) | 49 |  |
|  | CCC (P) => TTC (F) | 6 |  |
|  | CCT (P) => CTT (L) | 21 |  |
|  | GCG (A) => GTG (V) | 9 |  |
|  | CCC (P) => CTC (L) | 9 |  |
|  | CTC (L) => TTC (F) | 5 |  |
|  | CCT (P) => TTT (F) | 14 |  |
|  | GCT (A) => GTT (V) | 2 |  |
|  | GCC (A) => GTC (V) | 1 |  |
| hydrophilic | CGT (R) => TGT (C) | 30 | 12.90% |
|  | CGC (R) => TGC (C) | 12 |  |
|  | CAT (H) => TAT (Y) | 18 |  |
|  | CAC (H) => TAC (Y) | 9 |  |
| hydrophobic-hydrophilic | CCC (P) => TCC (S) | 9 | 7.29% |
|  | CCA (P) => TCA (S) | 8 |  |
|  | CCT (P) => TCT (S) | 19 |  |
|  | CCG (P) => TCG (S) | 3 |  |
| hydrophilic-hydrophobic | TCG (S) => TTG (L) | 51 | 48.22% |
|  | TCA (S) => TTA (L) | 78 |  |
|  | TCT (S) => TTT (F) | 48 |  |
|  | ACT (T) => ATT (I) | 4 |  |
|  | TCC (S) => TTC (F) | 34 |  |
|  | ACA (T) => ATA (I) | 4 |  |
|  | CGG (R) => TGG (W) | 32 |  |
|  | ACG (T) => ATG (M) | 6 |  |
|  | ACC (T) => ATC (I) | 1 |  |
| hydrophilic-stop | CAA (Q) => TAA (X) | 2 | 0.75% |
|  | CGA (R) => TGA (X) | 2 |  |

**Supplementary Table 8.** The abbreviations and NCBI accession numbers of mitochondrial genomes used in this study

| **Species** | **Abbreviations** | **Accession Numbers** |
| --- | --- | --- |
| *Hevea brasiliensis* | *H.brasiliensis* | AP_014526.1 |
| *Salix brachista* | *S.brachista* | CM_018591.1 |
| *Zea mays subsp. mays* | *Z.mays* | CM_025451.1 |
| *Arabidopsis thaliana* | *A.thaliana* | JF_729201.1 |
| *Daucus carota subsp. sativus* | *D.sativus* | JQ_248574.1 |
| *Glycine max* | *G.max* | JX_463295.1 |
| *Ginkgo biloba* | *G.biloba* | KM_672373.1 |
| *Nymphaea colorata* | *N.colorata* | KY_889142.1 |
| *Citrus sinensis* | *C.sinensis* | MG_736621.1 |
| *Eucalyptus grandis* | *E.grandis* | MG_925370.1 |
| *Solanum tuberosum* | *S.tuberosum* | MN_104801.1 |
| *Nicotiana tabacum* | *N.tabacum* | MN_651324.1 |
| *Brassica rapa* | *B.rapa* | MT_409179.1 |
| *Brassica napus* | *B.napus* | NC_008285.1 |
| *Sorghum bicolor* | *S.bicolor* | NC_008360.1 |
| *Vitis vinifera* | *V.vinifera* | NC_012119.1 |
| *Helianthus annuus* | *H.annuus* | NC_023337.1 |
| *Utricularia reniformis* | *U.reniformis* | NC_034982.1 |
| *Platycodon grandiflorus* | *P.grandiflorus* | NC_035958.1 |
| *Solanum lycopersicum* | *S.lycopersicum* | NC_035963.1 |
| *Triticum aestivum* | *T.aestivum* | NC_036024.1 |
| *Manihot esculenta* | *M.esculenta* | NC_045136.1 |
| *Camellia sinensis var. Assamica cv.duntsa* | *C.duntsa* | OL_989850.1 |
| *Camellia sinensis* | *C.sinensis* | NC_037304.1 |

**Supplementary Table 9.** The genes information contained in locally collinear blocks in mitochondrial genomes of C.duntsa

| *C.duntsa position*  */start (bp) -end (bp)* | *Gene* |
| --- | --- |
| *2966-3268* | *nad4L* |
| *3454-4032* | *atp4* |
| *5875-6627* | *ccmC* |
| *68596-70092* | *cox1* |
| *71845-74117* | *ccmFC* |
| *90325-92292* | *matR* |
| *140035-140391* | *nad3* |
| *140440-140817* | *rps12* |
| *175640-176119* | *atp8* |
| *176972-177769* | *cox3* |
| *177697-178083* | *sdh4* |
| *203565-204395* | *rps4* |
| *205054-205671* | *nad6* |
| *388722-389168* | *rps7* |
| *416365-422998* | *nad7* |
| *443613-444218* | *rps1* |
| *599527-599847* | *sdh3* |
| *631169-632350* | *cob* |
| *633679-633981* | *rps14* |
| *633983-634546* | *rpl5* |
| *639179-639529* | *rps13* |
| *662071-662862* | *cox2* |
| *903982-904554* | *nad9* |
| *931601-931885* | *rps19* |
| *975761-981639* | *nad4* |
| *986298-987245* | *atp6* |
| *994997-995344* | *mttB* |
| *1004252-1004872* | *ccmB* |
| *1005157-1005645* | *rpl10* |
| *1034419-1036215* | *ccmFN* |
| *1057612-1059141* | *atp1* |

**Supplementary Table 10.**Protein-coding genes annotated in *C.duntsa* mitochondrial genome in comparison to related species

| gene | *C.duntsa* | C.sinensis | C.sinensis | C. sinensis var. Assamica |
| --- | --- | --- | --- | --- |
|  | OL989850 | NC043914 | OM809792 | MK574877 |
| atp1 | 1 | 1 | 1 | 1 |
| atp4 | 1 | 1 | 2 | 1 |
| atp6 | 1 | 1 | 1 | 1 |
| atp8 | 1 | 0 | 2 | 1 |
| atp9 | 3 | 1 | 1 | 2 |
| ccmB | 1 | 1 | 1 | 1 |
| ccmC | 1 | 1 | 2 | 1 |
| ccmFC | 1 | 1 | 1 | 1 |
| ccmFN | 1 | 1 | 1 | 1 |
| cob | 1 | 1 | 1 | 1 |
| cox1 | 1 | 0 | 1 | 1 |
| cox2 | 1 | 1 | 2 | 1 |
| cox3 | 1 | 0 | 1 | 1 |
| matR | 1 | 1 | 1 | 1 |
| mttB | 1 | 1 | 1 | 1 |
| nad1 | 1 | 1 | 1 | 2 |
| nad2 | 1 | 1 | 1 | 2 |
| nad3 | 1 | 1 | 1 | 1 |
| nad4 | 1 | 1 | 1 | 1 |
| nad4L | 1 | 1 | 2 | 1 |
| nad5 | 1 | 1 | 1 | 1 |
| nad6 | 1 | 1 | 1 | 1 |
| nad7 | 1 | 0 | 1 | 1 |
| nad9 | 1 | 0 | 1 | 2 |
| rpl10 | 1 | 1 | 1 | 1 |
| rpl16 | 2 | 0 | 1 | 1 |
| rpl2 | 2 | 0 | 0 | 1 |
| rpl5 | 1 | 1 | 1 | 1 |
| rps1 | 1 | 1 | 1 | 1 |
| rps12 | 1 | 1 | 1 | 1 |
| rps13 | 1 | 2 | 1 | 1 |
| rps14 | 1 | 1 | 1 | 1 |
| rps16 | 0 | 2 | 0 | 0 |
| rps19 | 4 | 0 | 2 | 2 |
| rps3 | 3 | 0 | 0 | 1 |
| rps4 | 1 | 0 | 1 | 1 |
| rps7 | 1 | 1 | 1 | 1 |
| sdh3 | 1 | 1 | 1 | 2 |
| sdh4 | 1 | 0 | 1 | 1 |

**Supplementary Table 11** NCBI accession numbers of mitochondrial genomes used in this study

| **Species** | **Family** | **Accession number** |
| --- | --- | --- |
| *Hevea brasiliensis* | *Euphorbiaceae* | AP_014526.1 |
| *Zea mays subsp. mays* | *Poaceae* | CM_025451.1 |
| *Arabidopsis thaliana* | *Cruciferae* | JF_729201.1 |
| *Glycine max* | *leguminousae* | JX_463295.1 |
| *Ginkgo biloba* | *Ginkgoaceae* | KM_672373.1 |
| *Eucalyptus grandis* | *Myrtaceae* | MG_925370.1 |
| *Solanum tuberosum* | *Solanaceae* | MN_104801.1 |
| *Nicotiana tabacum* | *Solanaceae* | MN_651324.1 |
| *Brassica rapa* | *Cruciferae* | MT_409179.1 |
| *Brassica napus* | *Cruciferae* | NC_008285.1 |
| *Helianthus annuus* | *Asteraceae* | NC_023337.1 |
| *Platycodon grandiflorus* | *Platycodon* | NC_035958.1 |
| *Solanum lycopersicum* | *Solanaceae* | NC_035963.1 |
| *Triticum aestivum* | *Poaceae* | NC_036024.1 |
| *Sorghum bicolor* | *Poaceae* | NC_008360.1 |
| *Camellia sinensis* | *Theaceae* | NC_043914.1 |
| *Manihot esculenta* | *Euphorbiaceae* | NC_045136.1 |
| *Camellia duntsa* | *Theaceae* | OL-989850.1 |
| *Arabidopsis thaliana* | *Cruciferae* | NC_037304.1 |
| *Camellia sinensis* | *Theaceae* | OM_809792.1 |
